# Metformin reveals a mitochondrial copper addiction of mesenchymal cancer cells

**DOI:** 10.1371/journal.pone.0206764

**Published:** 2018-11-06

**Authors:** Sebastian Müller, Antoine Versini, Fabien Sindikubwabo, Guillaume Belthier, Supaporn Niyomchon, Julie Pannequin, Laurence Grimaud, Tatiana Cañeque, Raphaël Rodriguez

**Affiliations:** 1 Chemical Biology of Cancer Team, Labellisée Ligue Contre le Cancer. PSL Research University, CNRS UMR3666 –INSERM U1143, Institut Curie, Paris, France; 2 IGF, University of Montpellier, CNRS–INSERM, Montpellier, France; 3 Sorbonne Universités, UPMC Université Paris 06, PSL Research University, CNRS UMR8640. Ecole Normale Supérieure, Paris, France; University of Alabama at Birmingham, UNITED STATES

## Abstract

The clinically approved drug metformin has been shown to selectively kill persister cancer cells through mechanisms that are not fully understood. To provide further mechanistic insights, we developed a drug surrogate that phenocopies metformin and can be labeled *in situ* by means of click chemistry. Firstly, we found this molecule to be more potent than metformin in several cancer cell models. Secondly, this technology enabled us to provide visual evidence of mitochondrial targeting with this class of drugs. A combination of fluorescence microscopy and cyclic voltammetry indicated that metformin targets mitochondrial copper, inducing the production of reactive oxygen species in this organelle, mitochondrial dysfunction and apoptosis. Importantly, this study revealed that mitochondrial copper is required for the maintenance of a mesenchymal state of human cancer cells, and that metformin can block the epithelial-to-mesenchymal transition, a biological process that normally accounts for the genesis of persister cancer cells, through direct copper targeting.

## Introduction

Metformin is a clinically approved biguanide drug used in the management of type 2 diabetes [1]. The observation that treatments with metformin reduced risks of cancers in diabetic patients has prompted the search for mechanisms through which this molecule operates in cancer cells [2, 3]. Metformin has been proposed to decrease glucose levels by activating AMP-activated protein kinase (AMPK) in hepatocytes resulting in a reduced activity of acetyl-CoA carboxylase and an induction of fatty acid oxidation [4]. Furthermore, it was shown that biguanides decrease the Krebs cycle [5] and inhibit complex I of the electron transport chain (ETC) as well as ATP synthase in cancer cells and in isolated mitochondria [6, 7]. Furthermore, inhibition of complex I of the ETC by metformin was shown to reduce tumorigenesis [8]. Although it has been proposed that metformin acts on mitochondria and alters metabolism in cancer cells [9], there is of yet no direct visual evidence that biguanides physically accumulate in mitochondria. Importantly, metformin and the structural variant phenformin selectively target persister cancer cells [5] and synergize with conventional chemotherapy [10, 11], through mechanisms that remain poorly understood. Cancer cells can undergo phenotypic alterations reminiscent of that observed during normal embryogenic development by going through epithelial-to-mesenchymal transition (EMT), to acquire physical properties allowing these cells to detach from primary sites and give rise to distal secondary tumors. Cells able to adopt such a metastable state have been shown to be refractory to conventional chemotherapies and these persister cancer cells have thus been associated to cancer relapse. In light of their activity in cancer, it is conceivable that biguanides directly alter EMT. Furthermore, biguanides interact with metals *in vitro* including copper [12, 13], hinting towards a hypothesis whereby formation of metal complexes in cells could directly affect biological processes reliant on these metals, putative mechanistic targets of biguanides.

To investigate the subcellular localization of metformin in an unbiased manner, we designed a surrogate drug that could be labeled with a fluorophore in cells using click chemistry. A similar strategy was instrumental in identifying lysosomal iron as a mechanistic target of salinomycin in models of mesenchymal breast cancer cells, thereby illuminating a role of iron in the maintenance of these cells [14]. Using this approach, we identified mitochondria as a targeted organelle of metformin, providing unprecedented visual evidence of the accumulation of biguanides in this cellular compartment. Furthermore, we found that metformin directly impacts on mitochondrial copper levels in cancer cells, leading to mitochondrial stress and apoptosis. Importantly, we observed that mesenchymal cancer cells are addicted to copper and that metformin inhibits EMT in these cells. Taken together, our findings reveal mitochondrial copper as a druggable target in cancer cells that can be exploited to prevent the genesis of mesenchymal cancer cells.

## Materials and methods

### Reagents

Copper chloride (CuCl_2_, 459097, Sigma Aldrich), Ferrostatin-1 (Fer-1, SML0583, Sigma Aldrich, 10 μM for 72 h), human epidermal growth factor (EGF, 130-093-825, Miltenyi Biotech, 100 ng/mL for 72 h), carbonyl cyanide *m*-chlorophenylhydrazone (CCCP, 50 μM), Metformin (1,1-Dimethylbiguanide hydrochloride, J63361, Alfa Aesar, 12 mM unless stated otherwise), Metforminyn (Met, in-house drug, 2 mM unless stated otherwise), Necrostatin-1 (N9037, Sigma Aldrich, 20 μM for 72 h), Oncostatin M (OSM, 295-OM-010, R&D, 100 ng/ml for 72 h), TGF-*β* (TGF-*β*, D1183, Selleckchem, 15 ng/ml for 72 h), DyLight 488 Phalloidin (12935S, Cell Signaling Technology, IF 1:40), Trypan blue solution 0.4% (EVE Cell Counting Slides, NanoEnTek kit, #E1020), Turn-off Cu^2+^ probe [24] (10 μM for 1 h for FACS and IF experiments), Turn-on Cu^+^ probe [25] (1 μM for 30 min for FACS experiments), Turn-off Fe^2+^ probe [26] (1 μM for 1h for FACS experiments), Z-VAD-FMK (550377, BD Biosciences, 50 μM for 72 h).

### Antibodies

5' AMP-activated protein kinase subunit α (AMPKα, 2532, Cell Signaling, WB 1:1000), AMPKα phosphorylated Thr172 (phospho-AMPKα-Thr172, 2535, Cell Signaling, WB 1:1000), Caspase 3 (9665S, Cell Signaling Technology, WB 1:500), CD133-PE (130-090-853, MACS Myltenyi Biotec, FC 1:100), CD24-APC (2155590, Sony Biotechnology Inc., FC 1:100), CD44-Alexa-Fluor-647 (NB500-481AF647, Novus Biologicals, FC 1:1000), CD44-PE (FAB4948P, R&D Systems, FC 1:100), Copper uptake protein 1 (Ctr1, ab129067, Abcam, WB 1:1000 or FC 1:100), Cytochrome c (12963, Cell Signaling Technology, IF 1:500), Cytochrome c oxidase subunit 4 (Cox4, ab16056, Abcam, WB 1:2000), E-cadherin (20023195, Cell Signaling Technology, WB 1:1000), E-cadherin (610181, BD Biosciences, IF 1:200), Fibronectin (F0791, Sigma Aldrich, WB 1:1000), Glucose transporter 1 (GLUT1, 07–1401, Upstate Millipore, WB 1:1000), Glyceraldehyde 3-phosphate dehydrogenase (GAPDH, 2118, Cell Signalling Technologies, WB 1:1000), Slug (9585S, Cell Signaling Technology, WB 1:500), Insulin-like growth factor 1 phosphorylated Tyr1131/Tyr1146 (IGF-1-P, 3021, Cell Signaling Technologies, WB 1:1000), Snail (3895, Cell Signaling Technology, WB 1:500), Superoxide dismutase 1 (SOD1, ab13498, Abcam, WB 1:1000), γ-Tubulin (T5326, Sigma Aldrich, WB 1:2000), Twist (sc-81417, Santa Cruz, WB 1:200), Vimentin (3932, Cell Signaling Technology, WB 1:500, 5741S, Cell Signaling Technology, WB 1:500), Zeb1 (sc-81428, Santa Cruz Biotechnology, WB 1:500), Zeb2 (sc-271984, Santa Cruz, WB 1:200). Secondary *antibodies for WB*: HRP anti-Mouse (A90-116P, Bethyl Laboratories, WB 1:30000) and HRP anti-Rabbit (A120-108P, Bethyl Laboratories, 1:30000). *Secondary antibodies for IF and FC*: Alexa Fluor 488 conjugate (A-11017 Mouse, A-11008 Rabbit, Life Technologies, IF 1:500), Alexa Fluor 594 conjugate (A-11032 Mouse, A-11072 Rabbit, Life Technologies, IF 1:500), Alexa Fluor 647 conjugate (A-20991 Rabbit, A-20990 Mouse, Life Technologies, IF 1:500). All antibodies were diluted in blocking solution (2 or 5% BSA, 0.1% Tween-20/TBS).

### General information of chemical synthesis

All starting materials were purchased from commercial sources and used without further purification, or purified according to *Purification of Laboratory Chemicals* (Armarego, W.L.F., Chai, C.L.L. 5^th^ edition). Reactions were monitored by thin-layer chromatography (TLC) using TLC silica gel coated aluminum plates 60F-254 (Merck). Metforminyn was purified using a Combiflash RF+ Teledyne Isco system. ^1^H NMR spectra were recorded at 300 MHz and ^13^C NMR at 75 MHz at 298 K. Chemical shifts were reported as δ values are expressed in ppm using the residual non-deuterated solvent as internal standard and coupling constants (*J*) in Hz. The following abbreviations are used: s, singlet; t, triplet; td, triplet doublet. UPLC trace and electrospray mass spectrum were obtained from a Waters Acquity SQD2 UPLC-MS system. HRMS was measured with a Bruker maXis LC-QTOF.

### Synthesis of metforminyn (Met)

In a sealed tube, commercially available dicyandiamide (169 mg, 2.0 mmol) and *N*-methylbut-3-yn-1-amine (200 mg, 2.4 mmol) were suspended in 20 mL of xylene, then 2.5 mL of a 1 M aq. HCl solution were added. The tube was closed and heated at 140°C for 5 h. After this time, the reaction was cooled to RT and MeOH was added, then the solvent was removed under vacuum. The crude residue was purified by chromatography using a combiflash apparatus (DCM/MeOH, 10 to 20%) to afford Met as a white solid (97 mg, 24%). ^1^H NMR (300 MHz, MeOD) δ 3.60 (2H, t, *J* = 7.0 Hz), 3.09 (3H, s,), 2.50 (2H, td, *J* = 7.0, 3.0 Hz), 2.36 (1H, t, *J* = 3.0 Hz). ^13^C NMR (75 MHz, MeOD) δ 160.8, 160.5, 81.9, 71.4, 50.3, 36.7, 18.3. HRMS (ESI-TOF) calculated for C_7_H_14_N_5_ [M+H]^+^ 168.1244, found 168.1246.

### Cell lines

Dulbecco’s Phosphate-Buffered Saline (14190–094, 500 mL, Gibco), Dulbecco’s Modified Eagle Medium (DMEM)/F12 (31331–028, 500 mL, Gibco), DMEM GlutaMAX (61965059, Thermo Fisher Scientific), Fetal Bovine Serum (FBS, 10270–106, Gibco), Hydrocortisone (H0888, Sigma Aldrich), Insulin (I0516 or I9278, Sigma Aldrich), PEN-STREP (DE17-602E, BioWhittaker, Lonza), Puromycin dihydrochloride (A11138-02, Life Technologies). MCF-7 (ATCC, HTB-22), MDA-MB-231 (ATCC, HTB-26), MDA-MB-468 (ATCC, HTB-132), DU-145 (HTB-81) and circulating tumor cells were grown in DMEM supplemented with 10% FBS and 1% penicillin (100 U/mL), streptomycin (100 μg/ mL) and incubated at 37°C with 5% CO_2_. To induce EMT, cells were treated with epidermal growth factor EGF (100 ng/mL) (Miltenyi Biotec) for 72 h. The human mammary epithelial cell line infected with a retrovirus carrying hTERT, SV40 and the oncogenic allele *HrasV12*, named HMLER cells were a generous gift from A. Puisieux. HMLER cells were cultured in DMEM/F12 supplemented with 10% FBS, 10 μg/mL insulin, 0.5 μg/mL hydrocortisone, 10 ng/mL hEGF, and 0.5 μg/mL puromycin. A mycoplasma test was performed using PCR mycoplasma detection kit (G238, Applied Biological Materials).

### Cell viability

Cell viability was carried out by plating 1000 cells/well in 96-well plates using CellTiter-Blue viability assay according to the manufacturer’s protocol. Cells were treated as indicated for 72 h. CellTiter-Blue reagent (G8081, Promega) was added after 72 h treatment and cells were incubated for 1–2 h before recording fluorescence intensities (ex. 560/20 nm; em. 590/10 nm) using a Perkin Elmer Wallac 1420 Victor2 Microplate Reader. Trypan blue exclusion and cell viability measurements were performed using an EVE Automated Cell Counter (NanoEnTek, #E1000) and EVE Cell Counting Slides. 10 μL of cell suspension were mixed with 10 μL of 0.4% Trypan solution and a final volume of 10 μL of this mixture used to count cells and assess viability according to the manufacturer’s protocol.

### Cell imaging

For immunofluorescence, cells were blocked with 2% BSA, 0.2% Tween-20/PBS (blocking buffer) for 20 min at RT. Cover-slips were incubated with 50 to 100 μL of diluted primary antibodies in blocking buffer 1 h at RT. Cover-slips were then washed three times with blocking buffer and incubated as described above with the appropriate secondary antibodies for 1 h. Cover-slips were washed three times with PBS and mounted using Vectashield Mounting Medium with DAPI (H-1200, VECTOR Laboratories). Fluorescence images were acquired using a Deltavision real-time microscope (Applied Precision). 60×/1.4NA and 100×/1.4NA objectives were used for 2D and 3D acquisitions that were deconvoluted with SoftWorx (Ratio conservative—15 iterations, Applied Precision) and processed with ImageJ.

### Chemical labeling of Met in cells

Cells were cultured at ~80% confluence and were treated with 0.3 mM Met for 3h. Cell were fixed with formaldehyde (2% in PBS, 12 min) prior to permeabilization (Triton X-100, 0.1% in PBS, 5 to 10 min) and washed three times with 1% BSA/PBS. The click reaction cocktail was prepared from Click-iT EdU Imaging kits (C10337, Life Technologies) according to the manufacturer’s protocol. Briefly, mixing 430 μL of 1× Click-iT reaction buffer with 20 μL of CuSO_4_ solution, 1.2 μL Alexa Fluor azide, 50 μL reaction buffer additive (sodium ascorbate) to reach a final volume of ~500 μL. Cover-slips were incubated with the click reaction cocktail in the dark at RT for 30 min, then washed three times with PBS. Immunofluorescence was then performed as indicated.

### Western blotting

Cells were treated as indicated and then washed with PBS. Proteins were solubilized in Laemmli buffer containing benzonase (70664–3, VWR, 1:100) added. Extracts were incubated at 37°C for 1 h. Proteins were resolved using pre-cast protein gels (Biorad, Mini-PROTEAN TGX, 4–20%, Cat# 456–1095), a Trans-Blot Turbo Transfer System (Biorad, Cat# 1704150) and Trans-Blot Turbo transfer buffer (20% EtOH). Membranes were incubated with primary antibodies overnight in 5% BSA in PBS, 0.1% Tween-20. Primary antibodies were detected using horse-radish-peroxidase (HRP) conjugated secondary antibodies (Jackson Laboratories) and the Super Signal enhanced chemiluminiscent detection kit (Thermo Scientific, Super Signal West Pico PLUS, Cat# 34580). Signals were revealed using a Fusion Solo S Imaging System (Vilber).

### Flow cytometry

Metal-specific probes were synthesized as previously described. Cells were treated as indicated in the figures. For mitochondrial copper and iron probes, cells were incubated with the relevant probe prior to being analyzed by flow cytometry. Cells were trypsinized (TrypLE Express Enzyme, Life Technologies, 12605010) and washed twice with ice cold PBS. For antibodies, cells were suspended in ice cold PBS containing 2% FBS and 1 mM EDTA (incubation buffer) and incubated for 20 min at 4°C with the relevant antibody. Cells were then washed twice with ice cold PBS and suspended in incubation buffer prior to being analyzed by flow cytometry. For each condition, at least 100.000 cells were counted. Data were recorded on a BD Accuri C6 (BD Biosciences) and processed using Cell Quest (BD Biosciences) and FlowJo (FLOWJO, LLC) software.

### Cellular levels of copper and iron

Cells were incubated with the relevant probes as described in Reagents section. Cells were then analyzed either by fluorescence microscopy or flow cytometry as described in Cell Imaging and Flow Cytometry sections.

### Cyclic voltammetry

Cyclic voltammetry experiments were performed with a three-electrode cell under argon. Saturated Calomel Electrode (SCE) was used as reference, a steady Glassy Carbon (GC) electrode of diameter 3 mm was selected as working electrode and a Platinum wire as counter-electrode. All cyclic voltammograms were recorded at RT with a μ-autolab III from Metrohm using Nova software with a scan rate of 2 V/s. MeCN was used as a degassed HPLC grade from Carlo Erba. Water was mQ H_2_O. Solutions with copper were prepared with 0.3 M *n*Bu_4_NBF_4_ (180 mg) in MeCN (1.8 mL) and 200 μL of a 20 mM Cu(MeCN)_4_PF_6_ stock solution in MeCN (7.4 mg/ mL). Then 20 μL of a 400 mM metformin or Met stock solution in mQ H_2_O (or PBS) (66.2 mg/mL or 112.0 mg/mL respectively) was added. Other MeCN solutions were prepared with 0.3 M *n*Bu_4_NBF_4_ (200 mg) in MeCN (2 mL) and 20 μL of a 200 mM Fe(NO_3_)_3_ stock solution in mQ H_2_O (48.4 mg/mL), or/and 20 μL of a 400 mM metformin or Met stock solution in mQ H_2_O (66.2 mg/1 mL or 112.0 mg/mL respectively). All aqueous solutions were prepared with 0.3 M Na_2_SO_4_ (85.2 mg) in mQ H_2_O (2 mL) and 20 μL of 200 mM Fe(NO_3_)_3_ stock solution in mQ H_2_O (48.4 mg/mL), or/and 20 μL of a 400 mM metformin stock solution in mQ H_2_O (66.2 mg/mL).

### ROS levels

Mitochondrial ROS was measured using MitoSOX Red Mitochondrial Superoxide Indicator (M36008, Thermo Fisher Scientific) according to the manufacturer’s protocol. In brief, cells were treated for the indicated time with the red fluorescent dye, trypsinized, washed and resuspended in ice cold PBS containing 2% FBS and 1 mM EDTA prior to being analyzed by flow cytometry.

### Mitochondrial membrane potential

Measurements were performed using the MitoProbe JC-1 assay kit (M34152, Thermo Fischer Scientific) according to the manufacturer’s protocol. In brief, cells were treated with 2 μM JC-1 or CCCP (*m*-chlorophenylhydrazone) control for the indicated time. Cells were then trypsinized, washed and resuspended in ice cold PBS containing 2% FBS and 1 mM EDTA prior to being analyzed by flow cytometry.

### Cell death

Cells were treated as indicated in the figures. After treatment, cell death was quantified using Annexin V-FITC (A)/Propidium Iodide (PI) assay according to the manufacturer’s protocol (Annexin V-FITC apoptosis detection kit II, 556570, BD Pharmingen). Data were analyzed by a LSRFortessa flow cytometer (BD Biosciences, San Jose, CA) and processed using Cell Quest (BD Biosciences) and FlowJo (FLOWJO, LLC) software.

### ICP-MS analyses

Glass vials equipped with Teflon septa were cleaned with nitric acid 65% (VWR, Suprapur, 1.00441.0250), washed with ultrapure water (Sigma Aldrich, 000000001012620500) and dried. Cells were treated as indicated and harvested using trypsinization (TrypLE Express Enzyme, Life Technologies, 12605010) followed by two washes with DPBS (Life Technologies, 14190169). Cells were then transferred to the cleaned vials and this material was lyophilized. Samples were subsequently digested using nitric acid 65% (VWR, Suprapur, 1.00441.0250) overnight followed by heating to 80°C for 2 h. Samples were diluted with ultrapure water (Sigma Aldrich, 000000001012620500) to a total concentration of 0.475N nitric acid and transferred to metal-free centrifuge vials (VWR, 89049–172) for subsequent ICP-MS analyses. Cu concentrations were measured using an Agilent 7900 ICP-QMS in low-resolution mode. Sample introduction was achieved with a micro-nebulizer (MicroMist, 0.2ml/min) through a Scott spray chamber. Cu was measured using a collision-reaction interface with helium gas (5mL/min) to remove polyatomic interferences. Scandium and indium internal standards were injected after inline mixing with the samples to verify the absence of signal drift and matrix effects. A mix of certified standards was measured at concentrations spanning those of the samples to convert count measurements to concentrations in the solution. Uncertainties on sample concentrations are calculated using algebraic propagation of ICP-MS blank and sample counts uncertainties. Results were normalized by dry weight and cell number.

### Quantification and statistical analyses

All results are presented as means with their standard deviation (SD), unless stated otherwise. Data are representative of at least three independent biological replicates, unless stated otherwise. Unpaired *t*-tests were used to calculate significance unless stated otherwise. P values are defined as: * p ≤ 0.05, ** p ≤ 0.01, *** p ≤ 0.001, **** p ≤ 0.0001.

## Results and discussion

### Development of a potent clickable metformin surrogate

**Fig 1:**
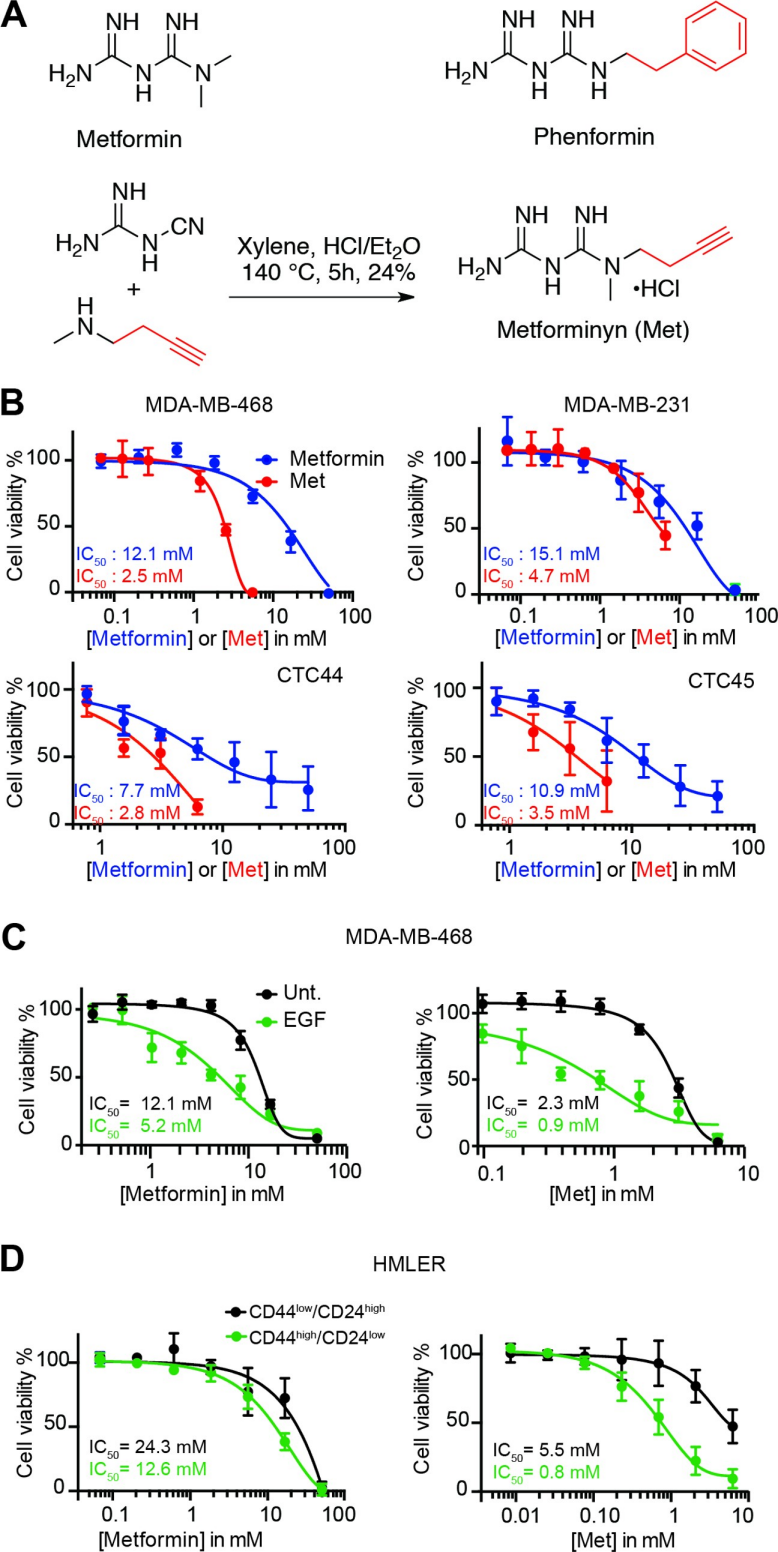
Metformin and more potent surrogate Met preferentially alter the proliferation of cancer cells in a mesenchymal state. (A) Molecular structures of metformin, phenformin and synthesis of Met. (B) Dose response viability curves of cancer cells treated as indicated for 72 h. (C) Dose response viability curves of cells in epithelial (dark) and mesenchymal (green) states of MDA-MB-468 cells treated as indicated for 72 h. (D) Dose response viability curves of HMLER cells in epithelial (dark) and mesenchymal (green) states treated as indicated for 72 h. Data points and error bars, mean values and SD of three independent biological replicates. Cells were treated at the IC_50_ concentration at 72 h of Metformin or Metforminyn respectively, unless stated otherwise. See also S1, S2, S3, S4 and S5 Figs.

We set out to develop a surrogate of metformin that would allow for direct visualization of the subcellular sites of action of biguanide drugs. To this end, we synthesized an alkyne-containing analogue we named metforminyn (Met) (**Figs 1 and S1**, **S2**, **S3 and S4**). This analogue was designed after metformin and phenformin, where distal methyl/benzyl substituents have been replaced by a terminal alkyne suitable for labeling in cells by means of click chemistry [15–19]. We anticipated that introducing a functional group at this position would lead to a small molecule probe that retains its activity against cancer cells. A comparative evaluation of metformin and Met against two triple negative human breast cancer cell lines, and circulating tumor cells (CTC) from patients with colorectal cancer [20], showed that the synthetic analogue exhibited a higher potency compared to metformin (**Fig 1**).

We then set out to investigate the effect of metformin and Met on cancer cells undergoing EMT. This process of EMT can be artificially induced from normal epithelial mammary cells or triple negative breast cancer cells, leading to cells with a pronounced mesenchymal phenotype [21, 22]. Importantly, we found that Met was more potent compared to metformin in these models, exhibiting a higher toxicity against MDA-MB-468 triple negative breast cancer cells transiently induced into a mesenchymal state using epidermal growth factor (EGF) [23] (**Fig 1**). A similar trend was observed for HMLER CD44^high^/CD24^low^ mesenchymal cells compared to their HMLER CD44^low^/CD24^high^ [21] epithelial counterpart (**Fig 1**).

To further investigate whether Met induced similar phenotypic alteration compared to metformin, we comparatively evaluated the effect of these molecules on levels of protein previously shown to be altered by metformin. Notably, we observed that metformin and Met increased levels of phosphorylated AMPKα-Thr172 and Glyceraldehyde 3-phosphate dehydrogenase (GADPH) whereas it decreased levels of GLUT1 (Glucose transporter 1) and the phosphorylated form of insulin-like growth factor 1 (IGF1 receptor-P) receptor (**S5 Fig**). Taken together, these data indicate that Met functionally phenocopied metformin in several models including mesenchymal cancer cells making it a suitable surrogate for subsequent cell imaging studies.

### Metformin targets mitochondrial copper

**Fig 2:**
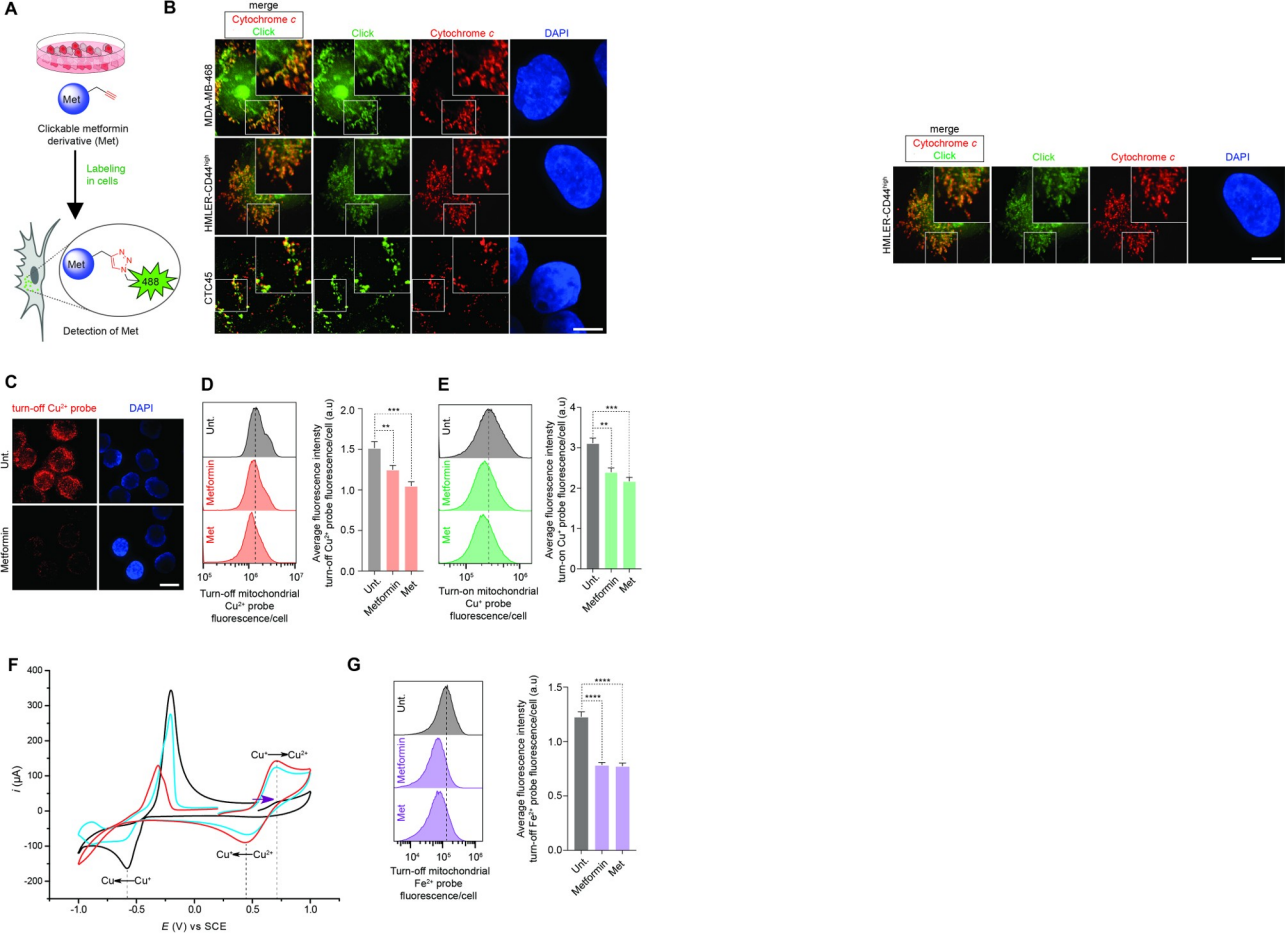
Biguanides directly target mitochondria and promote copper oxidation. (A) Schematic illustration of the labeling of Met in cells using click chemistry. (B) Fluorescence microscopy images of labeled Met (green). Cells were treated with Met and subjected to click-labeling as described in **Methods**. Mitochondria were detected using cytochrome *c* immunostaining (red) and 4',6-diamidino-2-phenylindole (DAPI) stains nuclear DNA (blue). Scale bar, 10 μm. (C) Fluorescence microscopy detection of Cu^2+^ in cancer cells treated as indicated for 48 h. Scale bar, 10 μm. (D) Flow cytometry analysis of mitochondrial Cu^2+^ in cancer cells treated as indicated for 48 h. Bar chart represents an average of three independent experiments. (E) Flow cytometry analysis of mitochondrial Cu^+^ in cancer cells treated as indicated for 48 h. Bar chart represents an average of three independent experiments. (F) Cyclic voltammetry measurements towards oxidation potentials (purple arrow) of a Cu^+^ solution. Data recorded in the absence (black) and presence of 2 mol equiv. metformin (blue) or 2 mol equiv. Met (red). Redox peak potentials are marked with dashed lines. (G) Flow cytometry analysis of Fe^2+^ in cancer cells treated as indicated for 48 h. MDA-MB-468 cells were used in Fig 2C, 2D, 2E and 2G and were treated as described in Methods. Cells were treated at the IC_50_ concentration at 72 h of Metformin or Metforminyn respectively, unless stated otherwise. See also S6, S7, S8, S9, S10 and S11 Figs. Bar chart represents an average of three independent experiments.

Cancer cells treated with Met were subjected to labeling in cells by means of click chemistry [14] to identify putative mechanistic sites of action of biguanides (**Fig 2**). In breast cancer cells, labeled Met co-localized with cytochrome *c*, a cellular component that uniquely characterizes mitochondria, along with weaker nuclear staining and larger *foci* that resembled that observed for nucleolar proteins (**Figs 2**and **S6**). In contrast, labeled Met could not be detected in the nucleus of CTC that responded to treatment (**Fig 2**), consistent with the idea that mitochondria are functional target organelles of biguanides. Remarkably, labeling of Met in cells, a chemical reaction that is normally catalyzed by Cu^+^, could be performed *in situ* without the need to incubate cells with additional copper catalyst (**S6 Fig**). Thus, the natural mitochondrial abundance of endogenous copper was sufficient to promote labeling of Met in native conditions. This provided further evidence that Met chemically interacted with copper in this organelle, in agreement with the previously reported capacity of metformin to form complexes with copper *in vitro* [12, 13]. Moreover, in agreement with metformin directly targeting mitochondria, fluorescence of labeled Met was significantly reduced upon co-treatment with excess metformin competitor (**S7 Fig**).

This finding encouraged us to investigate the effect of metformin and Met on the mitochondrial levels of Cu^2+^ using a selective turn-off fluorescent probe [24]. Metformin and Met induced a reduction in fluorescence of this turn-off probe in MDA-MB-468 cells, as defined by microscopy and fluorescence activated cell sorting (FACS), indicating increased levels of Cu^2+^ (**Fig 2**). Furthermore, we found that metformin and Met decreased mitochondrial Cu^+^ levels using a selective turn-on fluorescent probe [25] (**Fig 2**). Importantly, metformin altered the redox properties of copper as defined by the occurrence of cyclic voltammetry peaks characteristic of quasi-reversible couples (**Fig 2**), and treatment with metformin or Met led to reduced protein level of the copper uptake protein 1 (Ctr1) in these cells **(S8 Fig**). These data indicated that increased levels of mitochondrial Cu^2+^ occurred as a result of biguanides directly interacting with, and promoting oxidation of Cu^+^ in this organelle, rather than by increasing cellular uptake of copper. For instance, Met was more effective at promoting oxidation of Cu^+^ and preventing reduction of Cu^+^ compared to metformin. This indicated that Met is more effective in stabilizing copper ions compared to metformin, which was in line with the higher potency of this analogue in cell based assays compared to the parent molecule. It is noteworthy that metformin and Met also increased levels of mitochondrial Fe^2+^ as defined by flow cytometry using a selective turn-off probe (**Fig 2**) [26], and cyclic voltammetry showed that metformin and Met altered the redox properties of this metal, with metformin being more effective than Met (**S9 Fig**). These data supported a model whereby biguanides can inhibit complex I by hijacking iron from Fe-S clusters. Alternatively, Fe^3+^ may also be consumed, acting as an electron scavenger of Cu^+^ oxidation upon treatment with metformin. Consistent with metformin acting as a pro-oxidant of copper in mitochondria and altering the ionic balance of copper and iron, treatment of MDA-MB-468 cells with metformin or Met led to increased levels of reactive oxygen species (ROS) in this organelle accompanied by the reduction of mitochondrial membrane potentials and an alteration of mitochondrial morphology (**S10 Fig**). Furthermore, cell death induced by metformin or Met was rescued by the caspase 3 inhibitor Z-VAD-FMK but not the ferroptosis and necrosis inhibitors ferrostatin-1 and necrostatin-1 [27], respectively, which was in line with apoptotic cell death taking place as a result of mitochondrial dysfunction (**S11 Fig**) [28].

### Copper is an essential component of the epithelial-to-mesenchymal transition

**Fig 3:**
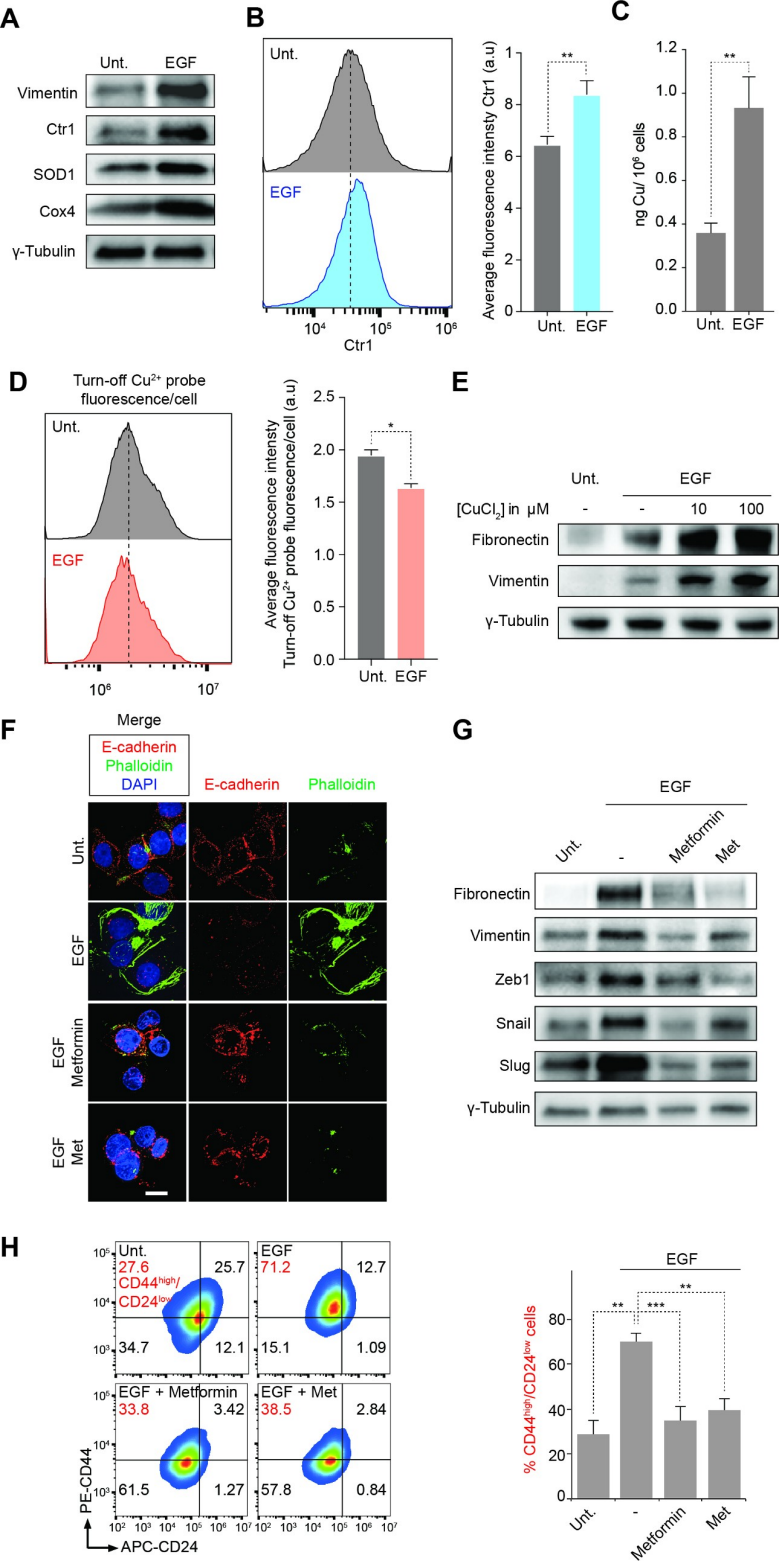
Copper is required to maintain a mesenchymal state of cancer cells. (A) Western blot analysis of levels of a mesenchymal marker (Vimentin), Ctr1 and copper-containing mitochondrial proteins (SOD1, Cox4) in cell treated with EGF for 72 h. (B) Flow cytometry analysis of Ctr1 protein level in cells treated with EGF for 72 h. (C) ICP-MS analysis of Cu content of cells treated with EGF for 72 h. Error bars represent the standard deviation of four independent analyses. (D) Flow cytometry analysis of Cu^2+^ in cells treated with EGF for 72 h. Bar chart represents an average of three independent experiments. (E) Western blot analysis of mesenchymal markers (Vimentin, Fibronectin) in cells treated as indicated for 72 h. (F) Fluorescence microscopy images of E-cadherin (red) and Phalloidin (green) in cells treated as indicated for 72 h. DAPI stains nuclear DNA (blue). Scale bar, 10 μm. (G) Western blot analysis of mesenchymal markers and EMT-TF in cancer cells treated as indicated for 72 h. (H) Flow cytometry analysis of cells surface markers of cancer cells treated as indicated for 72 h and corresponding quantification. Bars and error bars, mean values and SD of three independent biological replicates. MDA-MB-468 cells were used throughout the figure. Cells were treated at the IC_50_ concentration at 72 h of Metformin or Metforminyn respectively, unless stated otherwise. See also S12 and S13 Figs.

The pronounced effect of Met against mesenchymal cancer cells, accumulation of this biguanide in mitochondria and its effect on copper homeostasis in this organelle led us to investigate a putative dependency of mesenchymal cancer cells on mitochondrial copper. Western blot analysis revealed that EGF-induced EMT led to a significant increase of copper-containing proteins including superoxide dismutase 1 (SOD1) and cytochrome *c* oxidase subunit 4 (Cox4), along with increased levels of copper transporter 1 (Ctr1) (**Fig 3**). We also observed an increase of mitochondrial copper and total copper levels in EGF-treated samples using a combination of FACS and inductively coupled plasma mass spectrometry (ICP-MS), respectively (**Fig 3**). Consistent with previous findings showing that mitochondrial load is higher in persister cancer cells [29, 30], these data suggested that this cell state requires higher needs for energy production, a biological process that heavily relies on metalloproteins and metal co-factors essential to the mitochondrial ETC [31]. Importantly, addition of copper synergized with EGF treatments to shift MDA-MB-468 cancer cells toward a mesenchymal phenotype as defined by increased levels of mesenchymal markers (e.g. Vimentin, Fibronectin) and the proportion of cells harboring a CD44^high^/CD24^low^ cell surface marker pattern (**Figs 3**and **S12**). Importantly, we also observed that copper synergized with TGF-*β* treatment to shift HMLER CD44^low^/CD24^high^ cells and DU-145 prostate cancer cells toward a mesenchymal phenotype as defined by increased ratios of cells with a CD44^high^/CD24^low^ or CD44^high^/CD133^high^ cell surface marker pattern, respectively, demonstrating a general role of copper in the regulation of EMT in different systems (**S12 Fig**).

Finally, induction of EMT in MDA-MB-468 cells using EGF was prevented by metformin and Met as defined by the subcellular localization of E-cadherin and phalloidin staining, levels of mesenchymal markers fibronectin, vimentin and EMT-transcription factors (TF) Zeb1, Snail and Slug as well as the proportion of cells harboring a CD44^high^/CD24^low^ cell surface marker pattern (**Figs 3**and **S13**). Similarly, this effect of metformin was also observed in MCF-7 breast cancer and in DU-145 prostate cancer cells (**S13 Fig**). Alternatively, this data may also reflect a preferential cell killing of Met when cells are in a mesenchymal state. Taken together, these data reveal a dependency of mesenchymal cancer cells on mitochondrial copper, providing a rationale for the heightened sensitivity of these cells to biguanides.

## Conclusion

To provide insights into mechanisms underlying the effect of metformin in cancer cells undergoing EMT or cancer cells exhibiting a mesenchymal phenotype, we have developed a surrogate probe of this drug that can be chemically labeled in cells by means of click chemistry. This probe is both more potent than metformin in cancer models and enables the visual detection of sites of action of biguanides in fixed cells. We provide visual evidence that biguanides preferentially target mitochondria in several cancer cell lines including cancer cells isolated from patients. Furthermore, the fact that azide-alkyne labeling of Met can be performed in cells in absence of added copper catalyst and that metformin promotes oxidation of Cu^+^ *in vitro* validate mitochondrial copper as a putative mechanistic target of metformin. In line with this, the biologically more potent analogue Met alters copper redox potentials to a greater extent than metformin, whereas the latter preferentially affects that of iron *in vitro*. Importantly, we show that metformin impacts levels of mitochondrial Cu^2+^ and Fe^2+^, providing a mechanistic rationale underlying inhibition of the ETC and ATP-synthase and suggesting that effects on AMPK occurs downstream of mitochondrial targeting. This investigation shows that cancer cells in a mesenchymal state are more sensitive to biguanides compared to their epithelial state and that this effect relies on mitochondrial copper targeting. Increased levels of mitochondrial copper-containing and copper uptake proteins during EMT, together with the synergistic effect of copper and growth factors of cytokines to induce EMT, support a prevalent role of mitochondrial copper in the maintenance of a mesenchymal state of cancer cells. We show a central role of copper in the regulation of the EMT program, a process associated to the genesis of persister cancer cells, metastasis and cancer relapse. In this context, inhibition of EMT by biguanides strongly argues in favor of a cross-talk between mitochondria and the nucleus, potentially involving mitochondrial metabolites that could regulate EMT plasticity. Moreover, it is conceivable that in models of diabetes [32], metformin targets mitochondrial copper to promote mitochondrial dysfunction, forcing cells to switch to a lower yielding anaerobic mode. These findings strengthen the notion that cancer cells exhibiting a mesenchymal phenotype are susceptible to mitochondrial copper targeting, which paves the way toward the establishment of novel therapeutic strategies [33–36].

## Supporting information

S1 Fig ^1^H NMR spectrum of Met. (PDF)

S2 Fig ^13^C NMR spectrum of Met. (PDF)

S3 Fig UPLC trace analysis of purified Met. (PDF)

S4 Fig Electrospray mass spectrum of Met. (TIF)

S5 Fig Western blot analysis of GAPDH, GLUT1, IGF1 receptor-P, AMPKα, and P-AMPKα-Thr172 levels. MDA-MB-468 cells were treated with metformin or Met as indicated for 72 h.
(TIF)

S6 Fig Fluorescence microscopy imaging of labeled Met and competition experiments. Metforminyn was labeled (green) in cells in the presence or absence of Met, and with 100 equivalents of Metformin (0.3 mM Met and 30 mM metformin).
(TIF)

S7 Fig Fluorescence microscopy imaging of labeled Met. (A) Metforminyn was labeled (green) in cells in the presence of added copper catalyst. (B) Metforminyn was labeled (green) in cells in absence of added copper catalyst. The indicated cell lines were treated with Met prior to being subjected to click-labeling as described in Method Details. Mitochondria were detected using cytochrome *c* immunostaining or mitotracker (red), DAPI stains nuclear DNA (blue). Scale bars, 10 μm.
(TIF)

S8 Fig Western blot and flow cytometry analyses of Ctr1 levels. (A) Western blot analysis of Ctr1 levels in MDA-MB-468 cells treated as indicated for 72 h. (B) Flow cytometry analysis of Ctr1 levels in MDA-MB-468 cells treated as indicated for 72 h.
(TIF)

S9 Fig Cyclic voltammetry analysis of an iron(III) solution. Data recorded towards reduction potentials (purple arrow) in the absence (black) and presence of 2 mol. equiv metformin (blue) or 2 mol. equiv metforminyn (red). Redox peak potentials are marked with dashed lines.
(TIF)

S10 Fig Analysis of mitochondrial dysfunction. (A) Flow cytometry analysis of mitochondrial ROS in MDA-MB-468 cells treated as indicated for 48 h. (B) Quantification of flow cytometry data monitoring mitochondrial membrane potentials in MDA-MB-468 cells treated as indicated for 48 h. CCCP (carbonyl cyanide *m*-chlorophenylhydrazone) was used as positive control. Bars and error bars, mean values and SD of three independent biological replicates. (C) Fluorescence microscopy analysis of mitochondrial morphology in cells treated as indicated. MDA-MB-468 cells were treated as indicated for 48 h. Mitochondria were detected using cytochrome *c* immunostaining (grey), DAPI stains nuclear DNA (blue). Scale bars, 10 μm.
(TIF)

S11 Fig Flow cytometry and western blot analyses of apoptosis. (A) Quantification of flow cytometry data monitoring Annexin V-FITC (AN) and Propidium Iodide (PI) fluorescence in MDA-MB-468 cells treated as indicated for 72 h. Bars and error bars, mean values and SD of three biological replicates. (B) Western blot analysis of caspase 3 cleavage. MDA-MB-468 cells were treated as indicated for 72 h.
(TIF)

S12 Fig Flow cytometry analysis of mesenchymal phenotypes. (A) MDA-MB-468 breast cancer cells were treated with EGF and CuCl_2_ as indicated for 72 h. (B) Transformed human mammary epithelial HMLER CD44^low^/CD24^high^ (HMLER CD24^high^) cells were treated with TGF-*β* and CuCl_2_ as indicated for 72h. (C) DU-145 prostate cancer cells were treated with TGF-*β* and CuCl_2_ as indicated for 72 h. Bars and error bars, mean values and SD of three independent biological replicates.
(TIF)

S13 Fig Flow cytometry and western blot analyses of the effect of metformin on EMT. (A) Western blot analysis of mesenchymal markers and EMT-TF in MDA-MB-468 breast cancer cells treated as indicated for 72 h. (B) Bar chart of viable cells using Trypan blue exclusion of MDA-MB-468 breast cancer cells treated as indicated for 72h. (C) Flow cytometry analysis of cells surface markers of MCF-7 cells treated as indicated for 72 h and corresponding quantification. Bars and error bars, mean values and SD of three independent biological replicates. (D) Western blot analysis of mesenchymal markers and EMT-TF in MCF-7 breast cancer cells treated as indicated for 72 h. (E) Flow cytometry analysis of cells surface markers of DU-145 cells treated as indicated for 72 h and corresponding quantification. Bars and error bars, mean values and SD of three independent biological replicates. (F) Western blot analysis of mesenchymal markers and EMT-TF in DU-145 prostate cancer cells treated as indicated for 72 h.
(TIF)

S14 Fig Syntheses supporting information. (PDF)

## References

[1] Knowler WC, Barrett-Connor E, Fowler SE, Hamman RF, Lachin JM, Walker EA, et al Reduction in the incidence of type 2 diabetes with lifestyle intervention or metformin. N Engl J Med. 2002;346: 393–403. 10.1056/NEJMoa012512 . 11832527 PMC1370926

[2] Evans JM, Donnelly LA, Emslie-Smith AM, Alessi DR, Morris AD. Metformin and reduced risk of cancer in diabetic patients. BMJ. 2005;330: 1304–5. 10.1136/bmj.38415.708634.F7 . 15849206 PMC558205

[3] Pernicova I, Korbonits M. Metformin-mode of action and clinical implications for diabetes and cancer. Nat Rev Endocrinol. 2014;10: 143–56. 10.1038/nrendo.2013.256 . 24393785

[4] Zhou G, Myers R, Li Y, Chen Y, Shen X, Fenyk-Melody J, et al Role of AMP-activated protein kinase in mechanism of metformin action. J Clin Invest. 2001;108: 1167–74. 10.1172/JCI13505 . 11602624 PMC209533

[5] Janzer A, German NJ, Gonzalez-Herrera KN, Asara JM, Haigis MC, Struhl K. Metformin and phenformin deplete tricarboxylic acid cycle and glycolytic intermediates during cell transformation and NTPs in cancer stem cells. Proc Natl Acad Sci USA. 2014;111: 10574–9. 10.1073/pnas.1409844111 . 25002509 PMC4115496

[6] Owen MR, Doran E, Halestrap AP. Evidence that metformin exerts its anti-diabetic effects through inhibition of complex 1 of the mitochondrial respiratory chain. Biochem J. 2000;348: 607–14. . 10839993 PMC1221104

[7] Bridges HR, Jones AJ, Pollak MN, Hirst J. Effects of metformin and other biguanides on oxidative phosphorylation in mitochondria. Biochem J. 2014;462: 475–87. 10.1042/BJ20140620 . 25017630 PMC4148174

[8] Wheaton WW, Weinberg SE, Hamanaka RB, Soberanes S, Sullivan LB, Anso E, et al Metformin inhibits mitochondrial complex I of cancer cells to reduce tumorigenesis. Elife. 2014;3: e02242 10.7554/eLife.02242 . 24843020 PMC4017650

[9] Andrzejewski S, Gravel SP, Pollak M, St-Pierre J. Metformin directly acts on mitochondria to alter cellular bioenergetics. Cancer Metab. 2014;2: 12 10.1186/2049-3002-2-12 . 25184038 PMC4147388

[10] Hirsch HA, Iliopoulos D, Tsichlis PN, Struhl K. Metformin selectively targets cancer stem cells, and acts together with chemotherapy to block tumor growth and prolong remission. Cancer Res. 2009;69: 7507–11. 10.1158/0008-5472.CAN-09-2994 . 19752085 PMC2756324

[11] Pattabiraman DR, Weinberg RA. Tackling the cancer stem cells—what challenges do they pose? Nat Rev Drug Discov. 2014;13: 497–512. 10.1038/nrd4253 . 24981363 PMC4234172

[12] Zhu M, Lu L, Yang P, Jin X. Bis(1,1‐di-methyl-biguanido)copper(II) octahydrate. Acta Crystallographica Section E. 2004;58: m217–m9.

[13] Repiscak P, Erhardt S, Rena G, Paterson MJ. Biomolecular mode of action of metformin in relation to its copper binding properties. Biochemistry. 2014;53: 787–95. 10.1021/bi401444n . 24433134

[14] Mai TT, Hamai A, Hienzsch A, Cañeque T, Müller S, Wicinski J, et al Salinomycin kills cancer stem cells by sequestering iron in lysosomes. Nat Chem. 2017;9: 1025–33. 10.1038/nchem.2778 . 28937680 PMC5890907

[15] Kolb HC, Finn MG, Sharpless KB. Click Chemistry: Diverse Chemical Function from a Few Good Reactions. Angew Chem Int Ed. 2001;40: 2004–21. . 11433435 10.1002/1521-3773(20010601)40:11<2004::AID-ANIE2004>3.0.CO;2-5

[16] Rostovtsev VV, Green LG, Fokin VV, Sharpless KB. A stepwise huisgen cycloaddition process: copper(I)-catalyzed regioselective "ligation" of azides and terminal alkynes. Angew Chem Int Ed. 2002;41: 2596–9. 10.1002/1521-3773(20020715)41:14<2596::AID-ANIE2596>3.0.CO;2-4 . 12203546

[17] Tornoe CW, Christensen C, Meldal M. Peptidotriazoles on solid phase: [1,2,3]-triazoles by regiospecific copper(i)-catalyzed 1,3-dipolar cycloadditions of terminal alkynes to azides. J Org Chem. 2002;67: 3057–64. . 11975567 10.1021/jo011148j

[18] Sletten EM, Bertozzi CR. Bioorthogonal chemistry: fishing for selectivity in a sea of functionality. Angew Chem Int Ed. 2009;48: 6974–98. 10.1002/anie.200900942 . 19714693 PMC2864149

[19] Cañeque T, Müller S, Rodriguez R. Visualizing biologically active small molecules in cells using click chemistry. Nat Rev Chem. 2018;2: 202–2015.

[20] Grillet F, Bayet E, Villeronce O, Zappia L, Lagerqvist EL, Lunke S, et al Circulating tumour cells from patients with colorectal cancer have cancer stem cell hallmarks in ex vivo culture. Gut. 2017;66: 1802–10. 10.1136/gutjnl-2016-311447 . 27456153 PMC5595103

[21] Morel AP, Lievre M, Thomas C, Hinkal G, Ansieau S, Puisieux A. Generation of breast cancer stem cells through epithelial-mesenchymal transition. PLoS One. 2008;3: e2888 10.1371/journal.pone.0002888 . 18682804 PMC2492808

[22] Nieto MA, Huang RY, Jackson RA, Thiery JP. Emt: 2016. Cell. 2016;166: 21–45. 10.1016/j.cell.2016.06.028 . 27368099

[23] Lo HW, Hsu SC, Xia W, Cao X, Shih JY, Wei Y, et al Epidermal growth factor receptor cooperates with signal transducer and activator of transcription 3 to induce epithelial-mesenchymal transition in cancer cells via up-regulation of TWIST gene expression. Cancer Res. 2007;67: 9066–76. 10.1158/0008-5472.CAN-07-0575 . 17909010 PMC2570961

[24] Wang H-H, Xue L, Fang Z-J, Li G-P, Jiang H. A colorimetric and fluorescent chemosensor for copper ions in aqueous media and its application in living cells. New J Chem. 2010;34: 1239–42.

[25] Dodani SC, Leary SC, Cobine PA, Winge DR, Chang CJ. A targetable fluorescent sensor reveals that copper-deficient SCO1 and SCO2 patient cells prioritize mitochondrial copper homeostasis. J Am Chem Soc. 2011;133: 8606–16. 10.1021/ja2004158 . 21563821 PMC3106114

[26] Petrat F, Weisheit D, Lensen M, de Groot H, Sustmann R, Rauen U. Selective determination of mitochondrial chelatable iron in viable cells with a new fluorescent sensor. Biochem J. 2002;362: 137–47. . 11829750 10.1042/0264-6021:3620137 PMC1222370

[27] Conrad M, Angeli JP, Vandenabeele P, Stockwell BR. Regulated necrosis: disease relevance and therapeutic opportunities. Nat Rev Drug Discov. 2016;15: 348–66. 10.1038/nrd.2015.6 . 26775689 PMC6531857

[28] Green DR, Reed JC. Mitochondria and apoptosis. Science. 1998;281: 1309–12. . 9721092 10.1126/science.281.5381.1309

[29] Farnie G, Sotgia F, Lisanti MP. High mitochondrial mass identifies a sub-population of stem-like cancer cells that are chemo-resistant. Oncotarget. 2015;6: 30472–86. doi: 10.18632/oncotarget.5401 . 26421710 10.18632/oncotarget.5401 PMC4741545

[30] Lamb R, Bonuccelli G, Ozsvari B, Peiris-Pages M, Fiorillo M, Smith DL, et al Mitochondrial mass, a new metabolic biomarker for stem-like cancer cells: Understanding WNT/FGF-driven anabolic signaling. Oncotarget. 2015;6: 30453–71. doi: 10.18632/oncotarget.5852 . 26421711 10.18632/oncotarget.5852 PMC4741544

[31] Balaban RS, Nemoto S, Finkel T. Mitochondria, oxidants, and aging. Cell. 2005;120: 483–95. 10.1016/j.cell.2005.02.001 . 15734681

[32] Logie L, Harthill J, Patel K, Bacon S, Hamilton DL, Macrae K, et al Cellular responses to the metal-binding properties of metformin. Diabetes. 2012;61: 1423–33. 10.2337/db11-0961 . 22492524 PMC3357267

[33] Laws K, Bineva-Todd G, Eskandari A, Lu C, O'Reilly N, Suntharalingam K. A Copper(II) Phenanthroline Metallopeptide That Targets and Disrupts Mitochondrial Function in Breast Cancer Stem Cells. Angew Chem Int Ed. 2017;57: 287–91. 10.1002/anie.201710910 . 29144008

[34] Skrott Z, Mistrik M, Andersen KK, Friis S, Majera D, Gursky J, et al Alcohol-abuse drug disulfiram targets cancer via p97 segregase adaptor NPL4. Nature. 2017;552: 194–9. 10.1038/nature25016 . 29211715 PMC5730499

[35] Weinberg SE, Chandel NS. Targeting mitochondria metabolism for cancer therapy. Nat Chem Biol. 2015;11: 9–15. 10.1038/nchembio.1712 . 25517383 PMC4340667

[36] Müller S, Cañeque T, Acevedo V, Rodriguez R. Targeting Cancer Stem Cells with Small Molecules. Isr J Chem. 2017;57: 239–50.

