# Correction: Metformin reveals a mitochondrial copper addiction of mesenchymal cancer cells

**DOI:** 10.1371/journal.pone.0208213

**Published:** 2018-11-26

**Authors:** Sebastian Müller, Antoine Versini, Fabien Sindikubwabo, Guillaume Belthier, Supaporn Niyomchon, Julie Pannequin, Laurence Grimaud, Tatiana Cañeque, Raphaël Rodriguez

Fig 2 is incorrect. The authors have provided a corrected version here.

**Fig 2:**
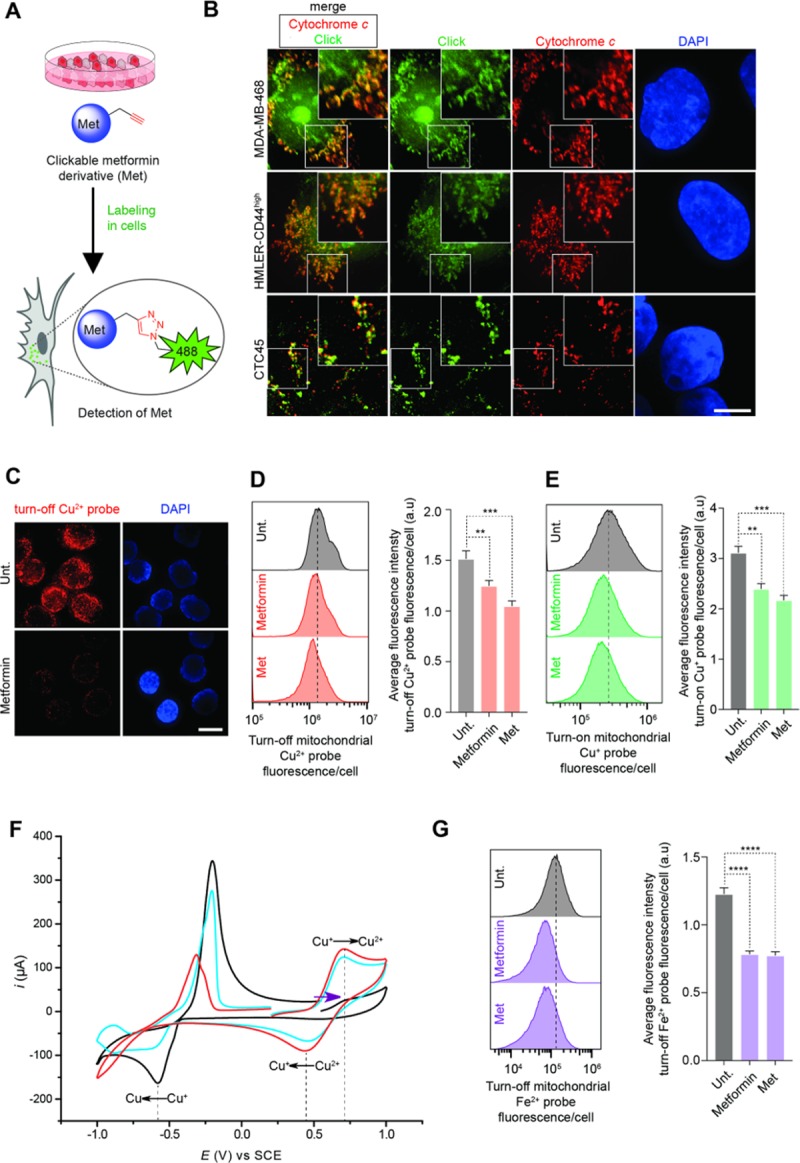
Biguanides directly target mitochondria and promote copper oxidation. (A) Schematic illustration of the labeling of Met in cells using click chemistry. (B) Fluorescence microscopy images of labeled Met (green). Cells were treated with Met and subjected to click-labeling as described in **Methods**. Mitochondria were detected using cytochrome *c* immunostaining (red) and 4',6-diamidino-2-phenylindole (DAPI) stains nuclear DNA (blue). Scale bar, 10 μm. (C) Fluorescence microscopy detection of Cu^2+^ in cancer cells treated as indicated for 48 h. Scale bar, 10 μm. (D) Flow cytometry analysis of mitochondrial Cu^2+^ in cancer cells treated as indicated for 48 h. Bar chart represents an average of three independent experiments. (E) Flow cytometry analysis of mitochondrial Cu^+^ in cancer cells treated as indicated for 48 h. Bar chart represents an average of three independent experiments. (F) Cyclic voltammetry measurements towards oxidation potentials (purple arrow) of a Cu^+^ solution. Data recorded in the absence (black) and presence of 2 mol equiv. metformin (blue) or 2 mol equiv. Met (red). Redox peak potentials are marked with dashed lines. (G) Flow cytometry analysis of Fe^2+^ in cancer cells treated as indicated for 48 h. MDA-MB-468 cells were used in Fig 2C, 2D, 2E and 2G and were treated as described in Methods. Cells were treated at the IC_50_ concentration at 72 h of Metformin or Metforminyn respectively, unless stated otherwise. See also S6, S7, S8, S9, S10 and S11 Figs. Bar chart represents an average of three independent experiments.
