# Supplementary figures and images for: Metformin reveals a mitochondrial copper addiction of mesenchymal cancer cells
Source: PLoS One. 2018 Nov 6;13(11):e0206764. doi: 10.1371/journal.pone.0206764 (PMC6219783; doi:10.1371/journal.pone.0206764)

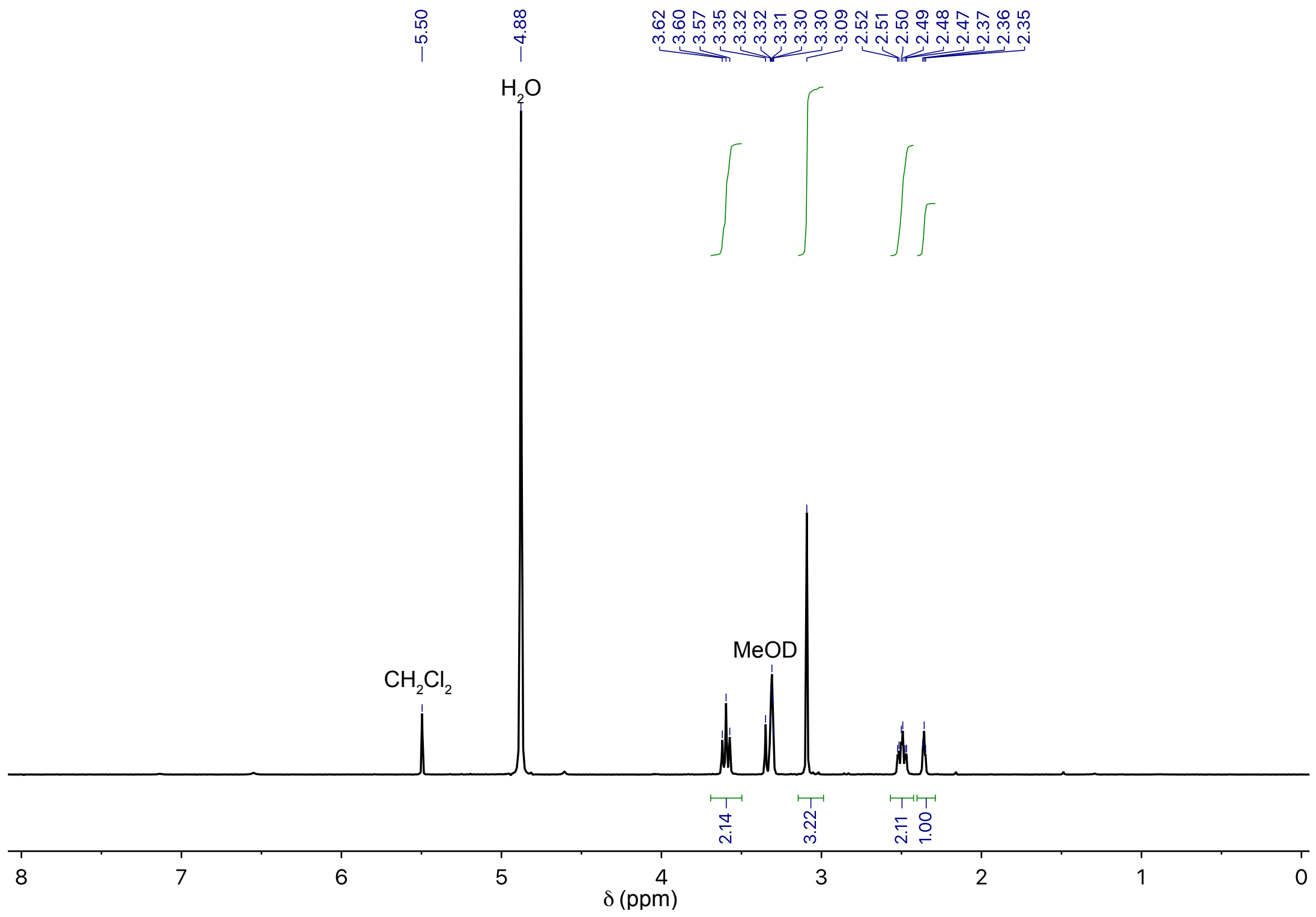

Supplement: S1 Fig — (PDF) [file pone.0206764.s001.pdf]

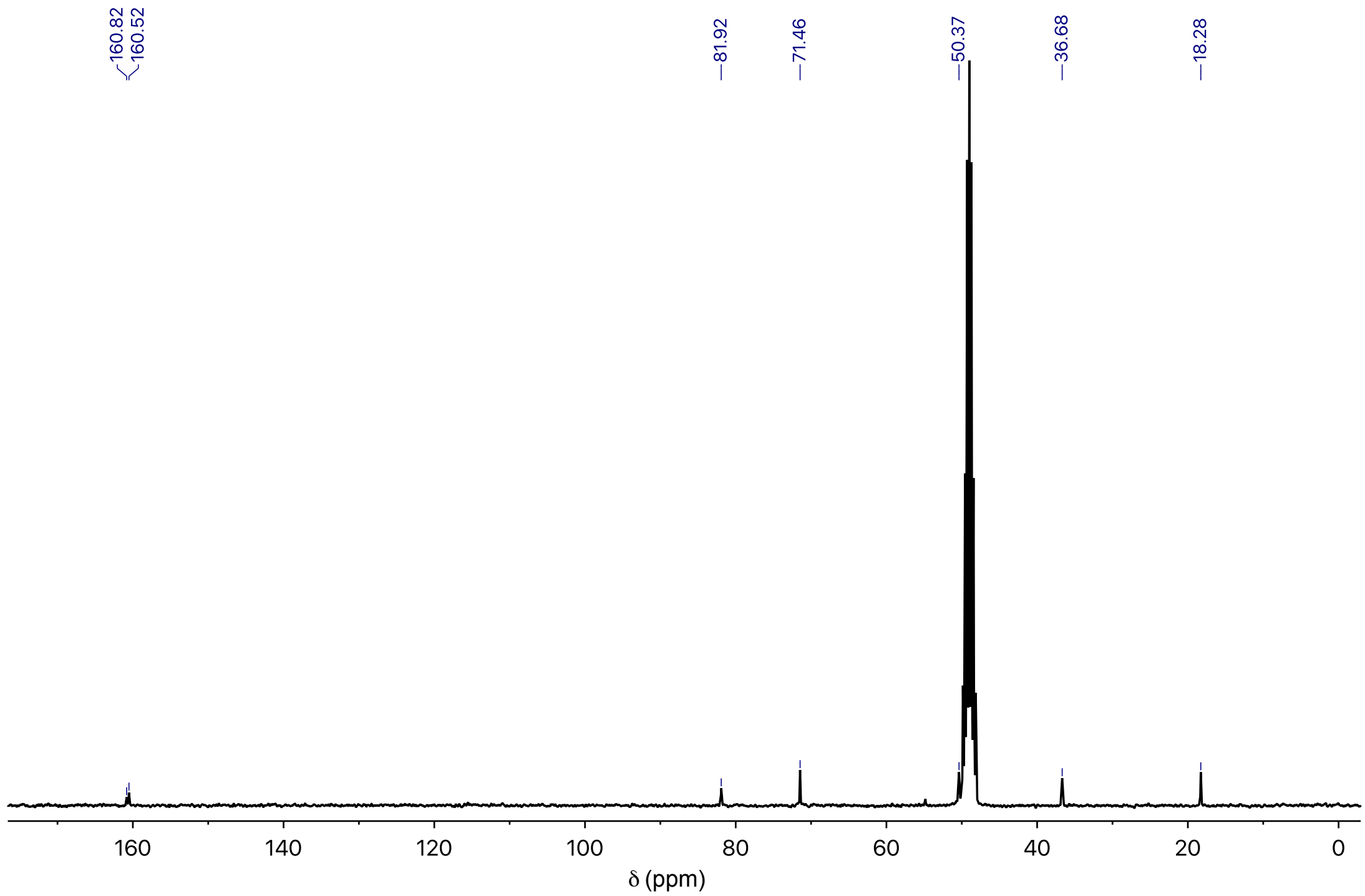

Supplement: S2 Fig — (PDF) [file pone.0206764.s002.pdf]

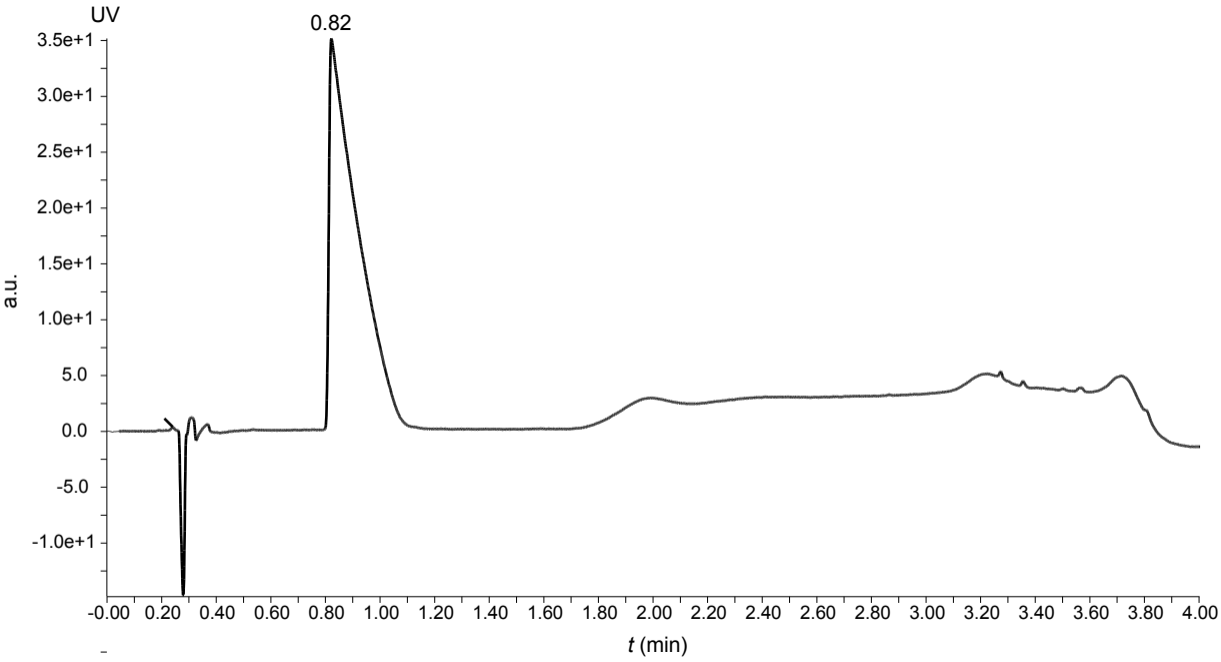

Supplement: S3 Fig — (PDF) [file pone.0206764.s003.pdf]

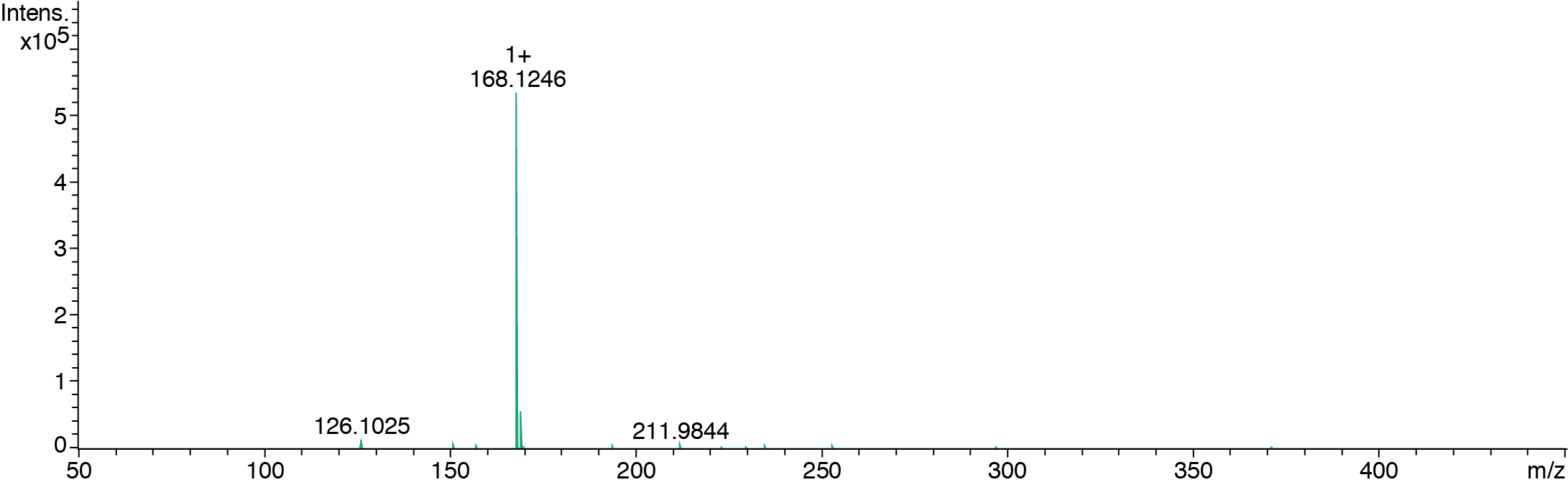

Supplement: S4 Fig — (TIF) [file pone.0206764.s004.tif]

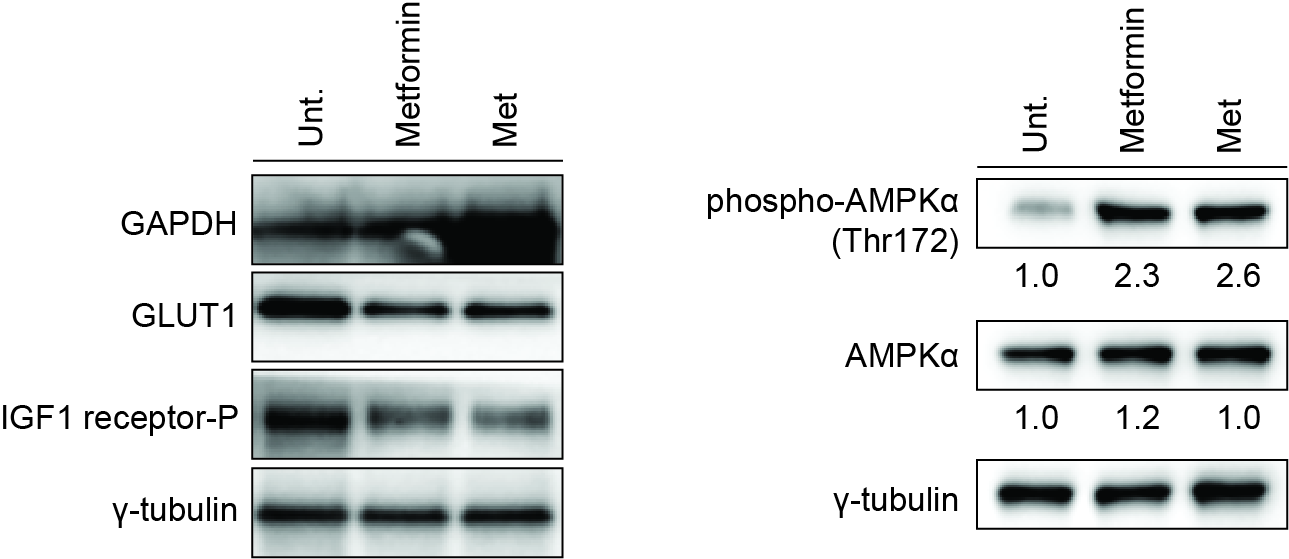

Supplement: S5 Fig — MDA-MB-468 cells were treated with metformin or Met as indicated for 72 h. (TIF) [file pone.0206764.s005.tif]

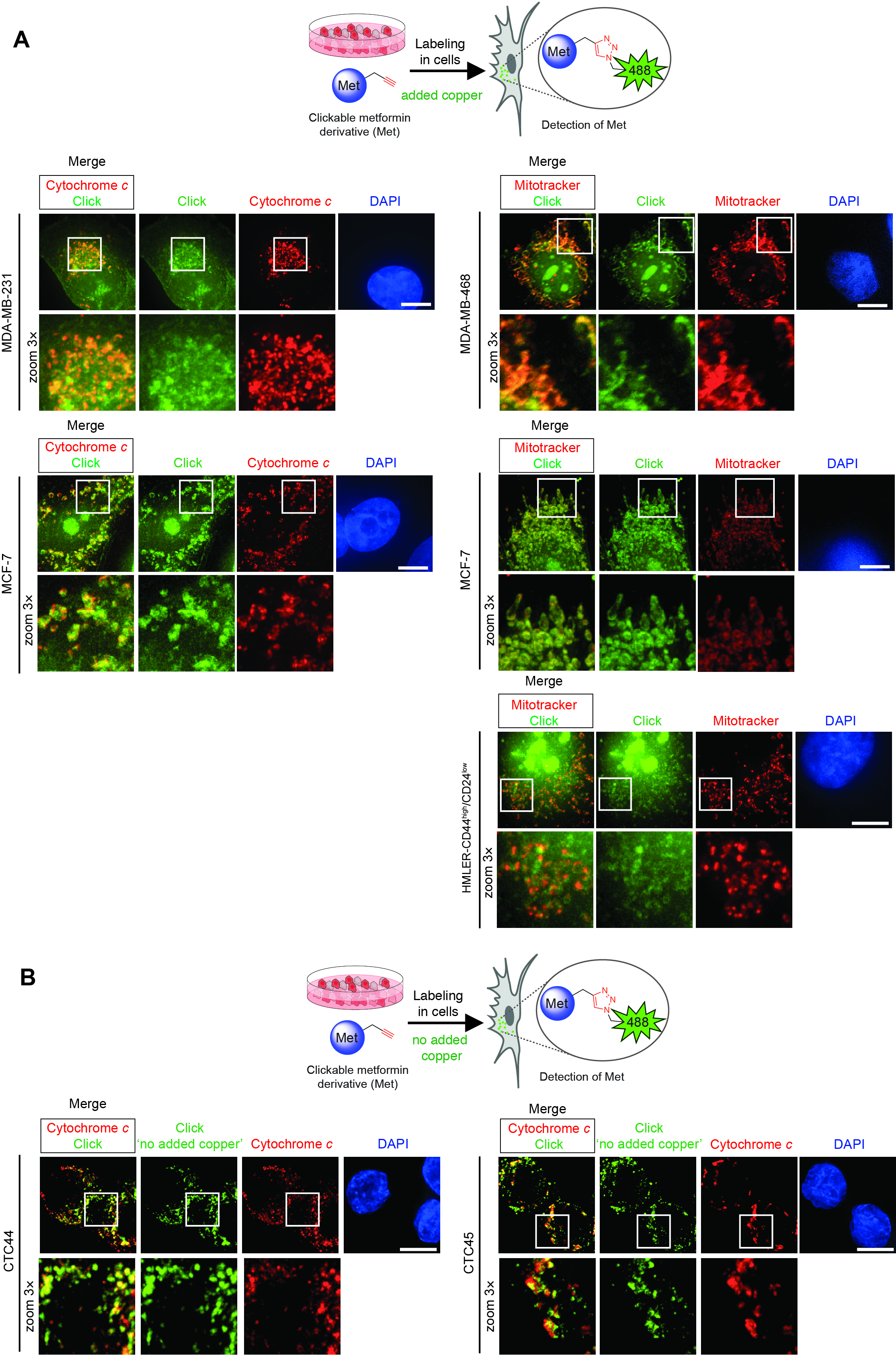

Supplement: S6 Fig — Metforminyn was labeled (green) in cells in the presence or absence of Met, and with 100 equivalents of Metformin (0.3 mM Met and 30 mM metformin). (TIF) [file pone.0206764.s006.tif]

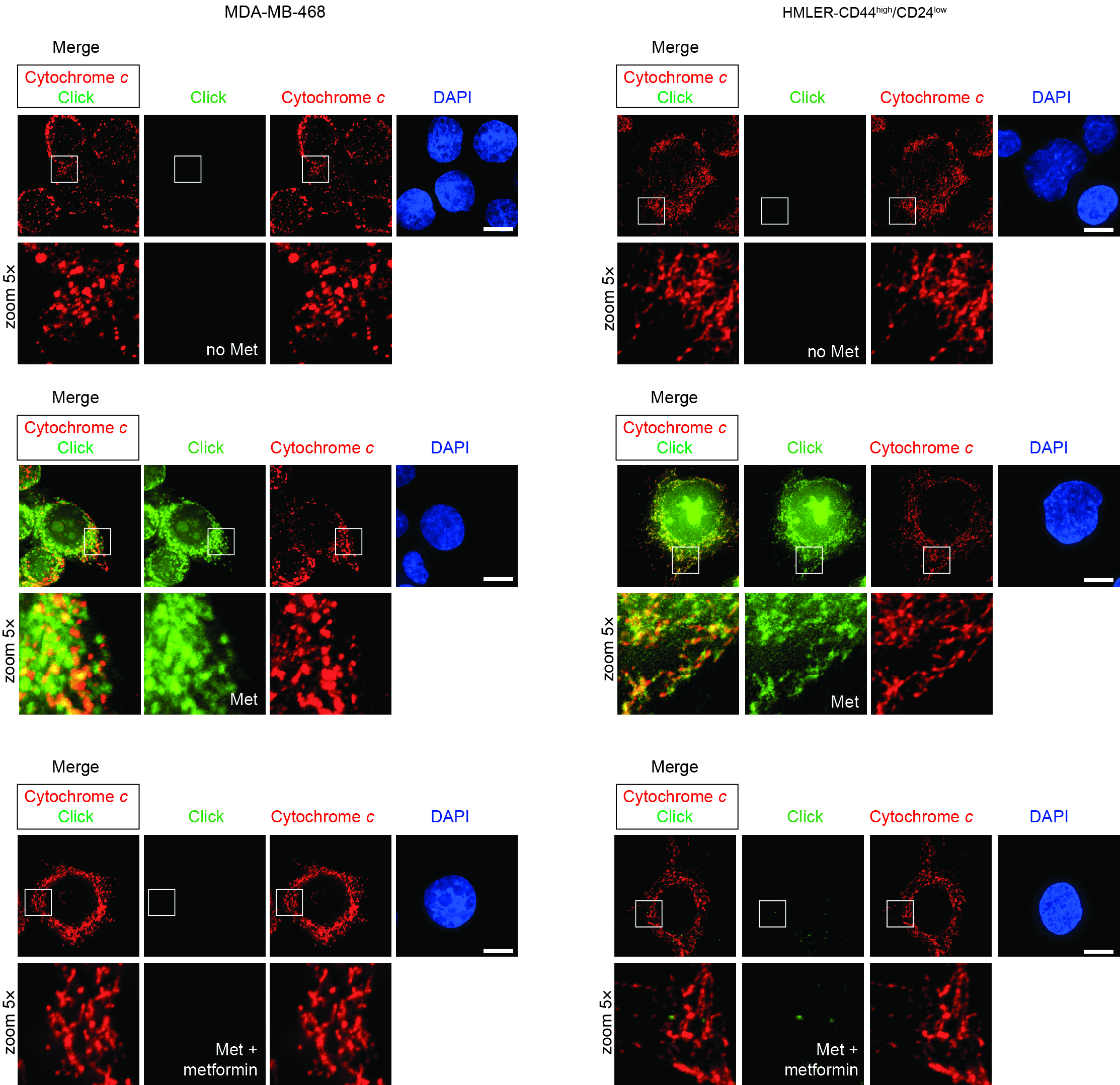

Supplement: S7 Fig — (A) Metforminyn was labeled (green) in cells in the presence of added copper catalyst. (B) Metforminyn was labeled (green) in cells in absence of added copper catalyst. The indicated cell lines were treated with Met prior to being subjected to click-labeling as described in Method Details. Mitochondria were detected using cytochrome c immunostaining or mitotracker (red), DAPI stains nuclear DNA (blue). Scale bars, 10 μm. (TIF) [file pone.0206764.s007.tif]

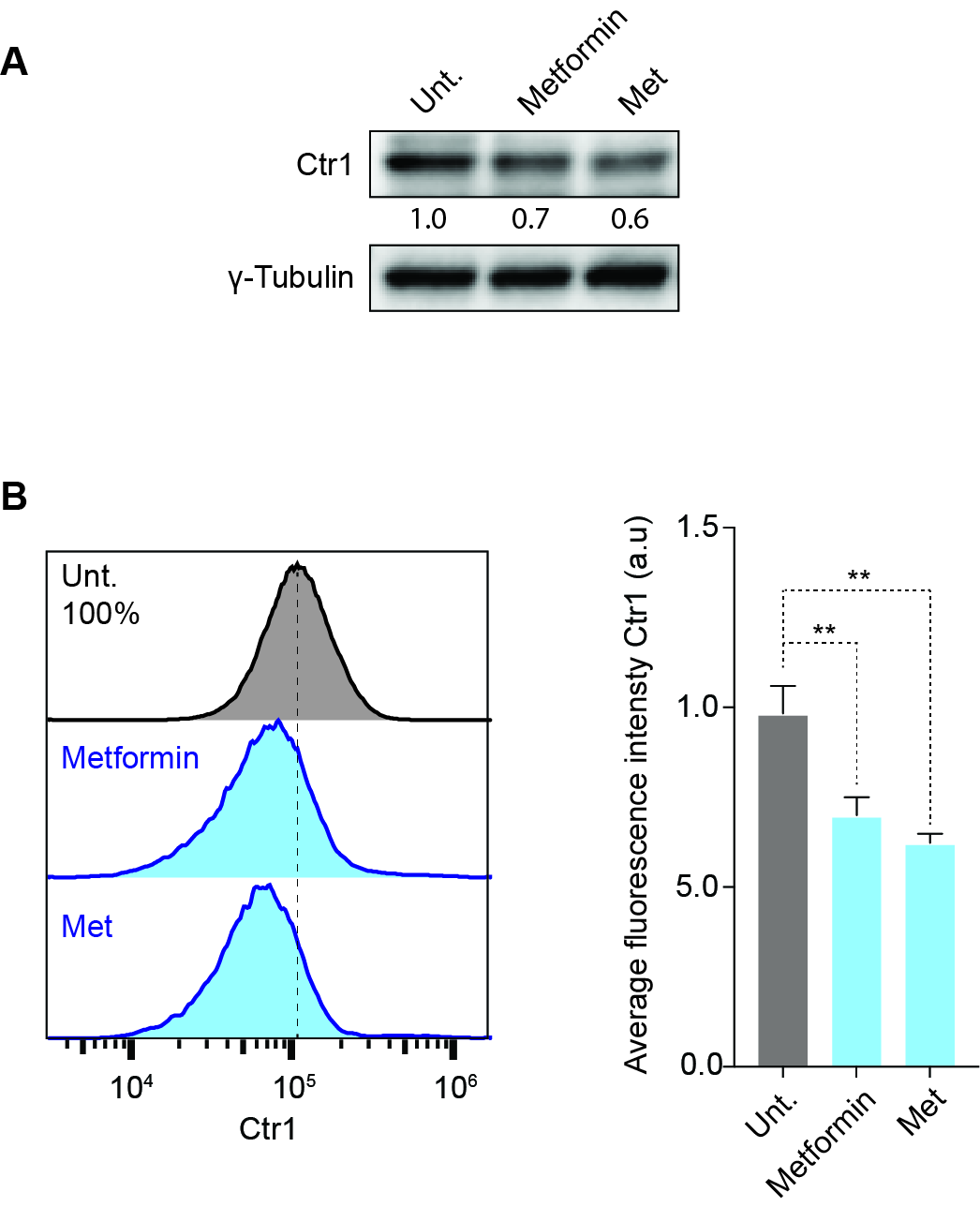

Supplement: S8 Fig — (A) Western blot analysis of Ctr1 levels in MDA-MB-468 cells treated as indicated for 72 h. (B) Flow cytometry analysis of Ctr1 levels in MDA-MB-468 cells treated as indicated for 72 h. (TIF) [file pone.0206764.s008.tif]

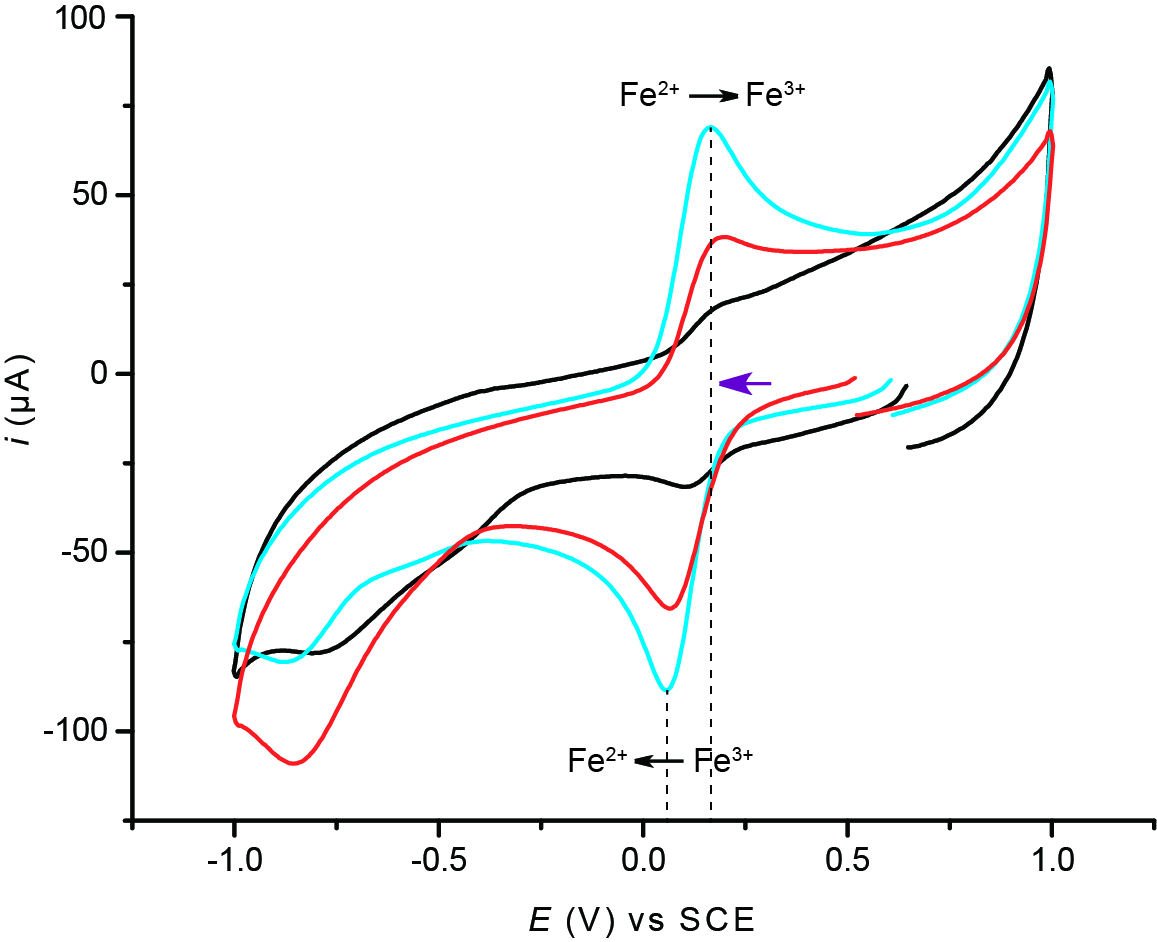

Supplement: S9 Fig — Data recorded towards reduction potentials (purple arrow) in the absence (black) and presence of 2 mol. equiv metformin (blue) or 2 mol. equiv metforminyn (red). Redox peak potentials are marked with dashed lines. (TIF) [file pone.0206764.s009.tif]

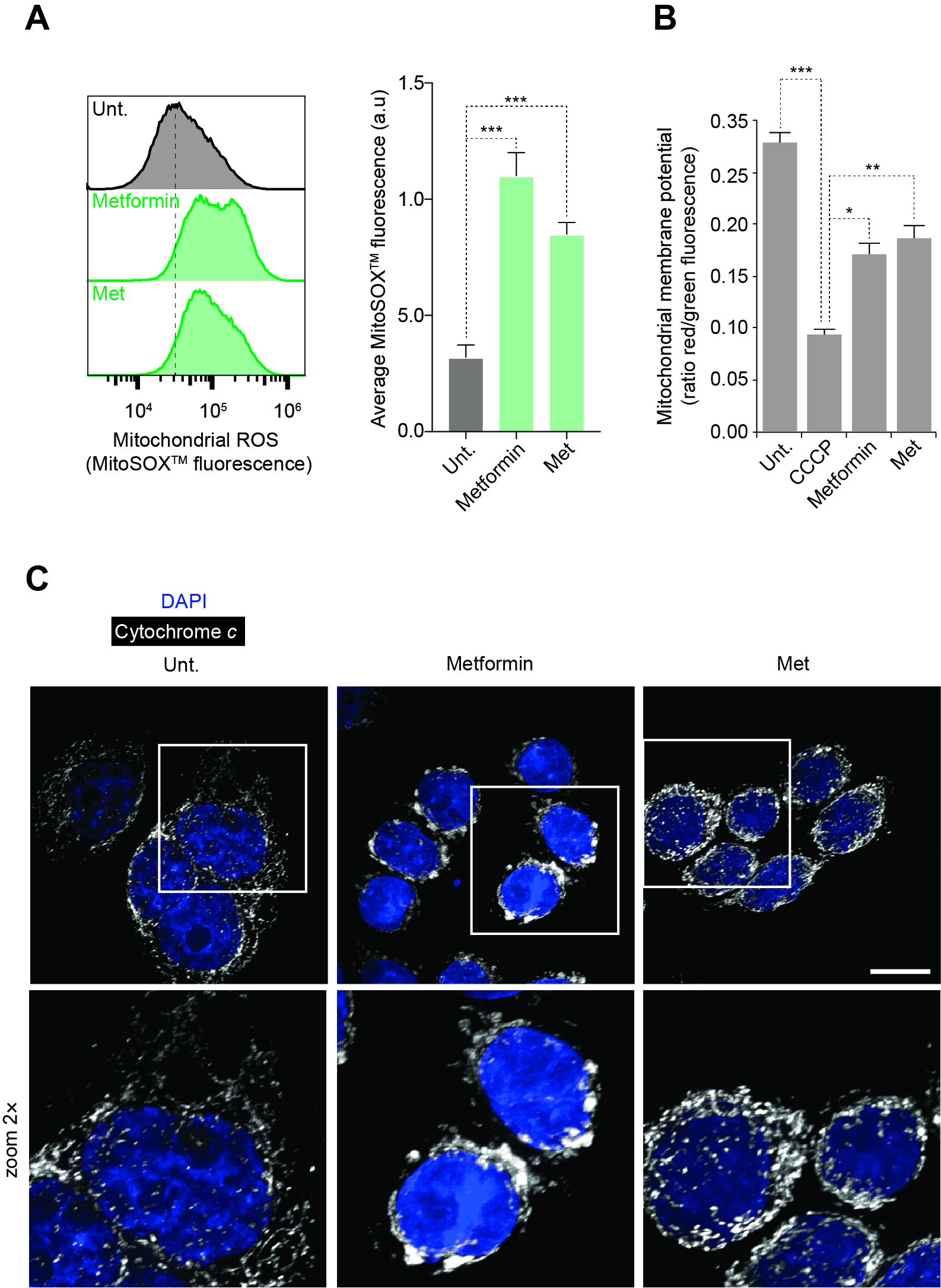

Supplement: S10 Fig — (A) Flow cytometry analysis of mitochondrial ROS in MDA-MB-468 cells treated as indicated for 48 h. (B) Quantification of flow cytometry data monitoring mitochondrial membrane potentials in MDA-MB-468 cells treated as indicated for 48 h. CCCP (carbonyl cyanide m-chlorophenylhydrazone) was used as positive control. Bars and error bars, mean values and SD of three independent biological replicates. (C) Fluorescence microscopy analysis of mitochondrial morphology in cells treated as indicated. MDA-MB-468 cells were treated as indicated for 48 h. Mitochondria were detected using cytochrome c immunostaining (grey), DAPI stains nuclear DNA (blue). Scale bars, 10 μm. (TIF) [file pone.0206764.s010.tif]

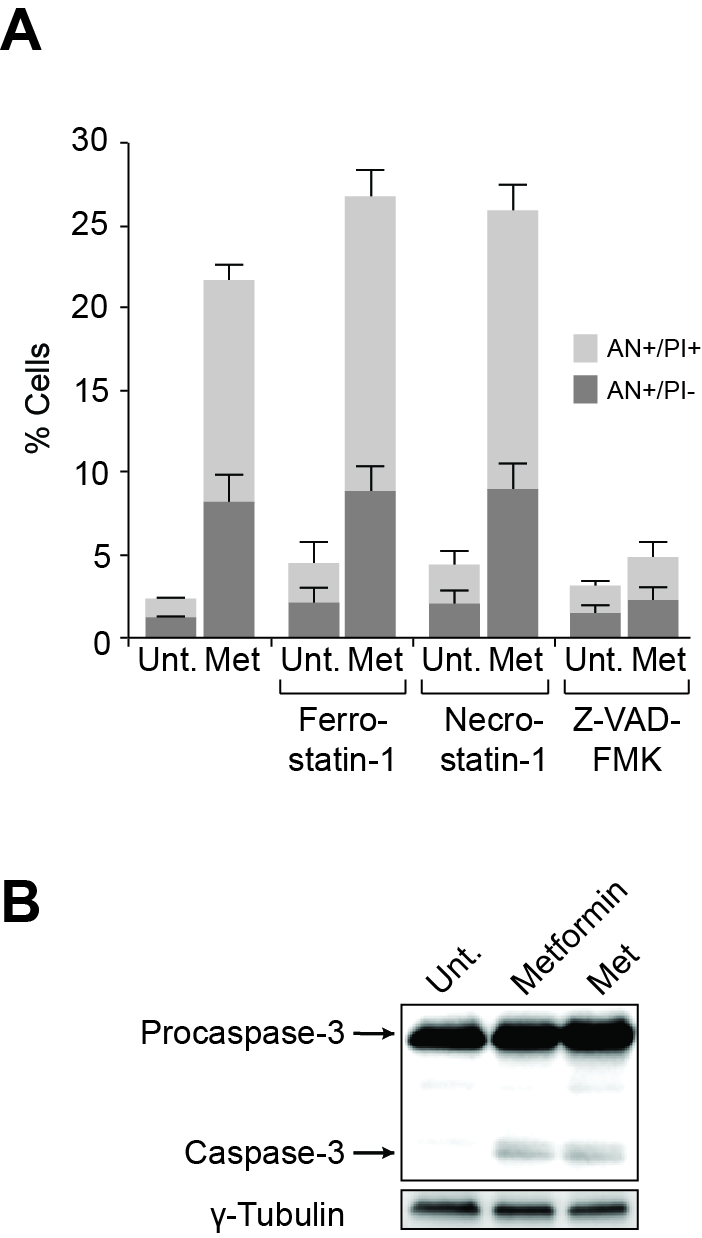

Supplement: S11 Fig — (A) Quantification of flow cytometry data monitoring Annexin V-FITC (AN) and Propidium Iodide (PI) fluorescence in MDA-MB-468 cells treated as indicated for 72 h. Bars and error bars, mean values and SD of three biological replicates. (B) Western blot analysis of caspase 3 cleavage. MDA-MB-468 cells were treated as indicated for 72 h. (TIF) [file pone.0206764.s011.tif]

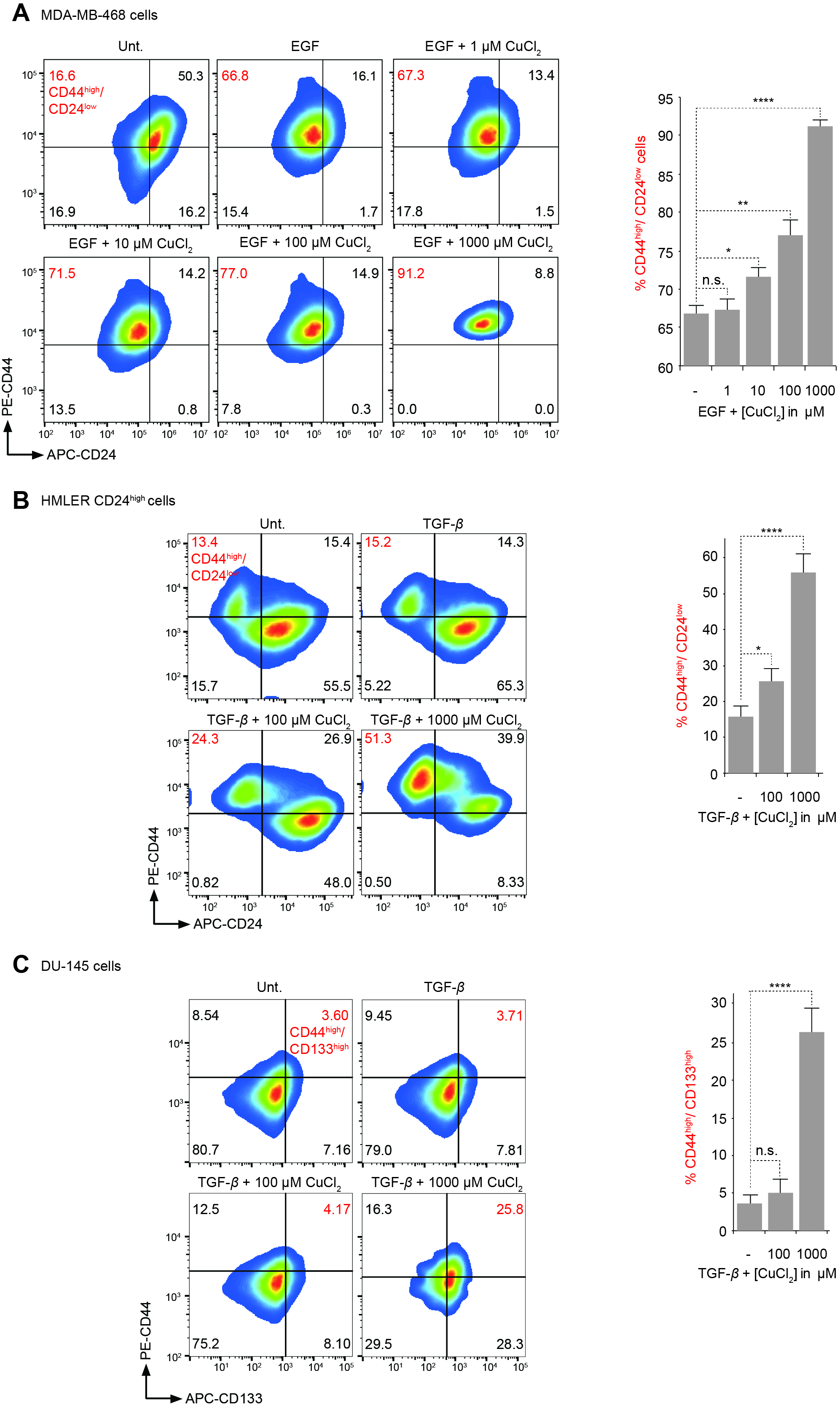

Supplement: S12 Fig — (A) MDA-MB-468 breast cancer cells were treated with EGF and CuCl2 as indicated for 72 h. (B) Transformed human mammary epithelial HMLER CD44low/CD24high (HMLER CD24high) cells were treated with TGF-β and CuCl2 as indicated for 72h. (C) DU-145 prostate cancer cells were treated with TGF-β and CuCl2 as indicated for 72 h. Bars and error bars, mean values and SD of three independent biological replicates. (TIF) [file pone.0206764.s012.tif]

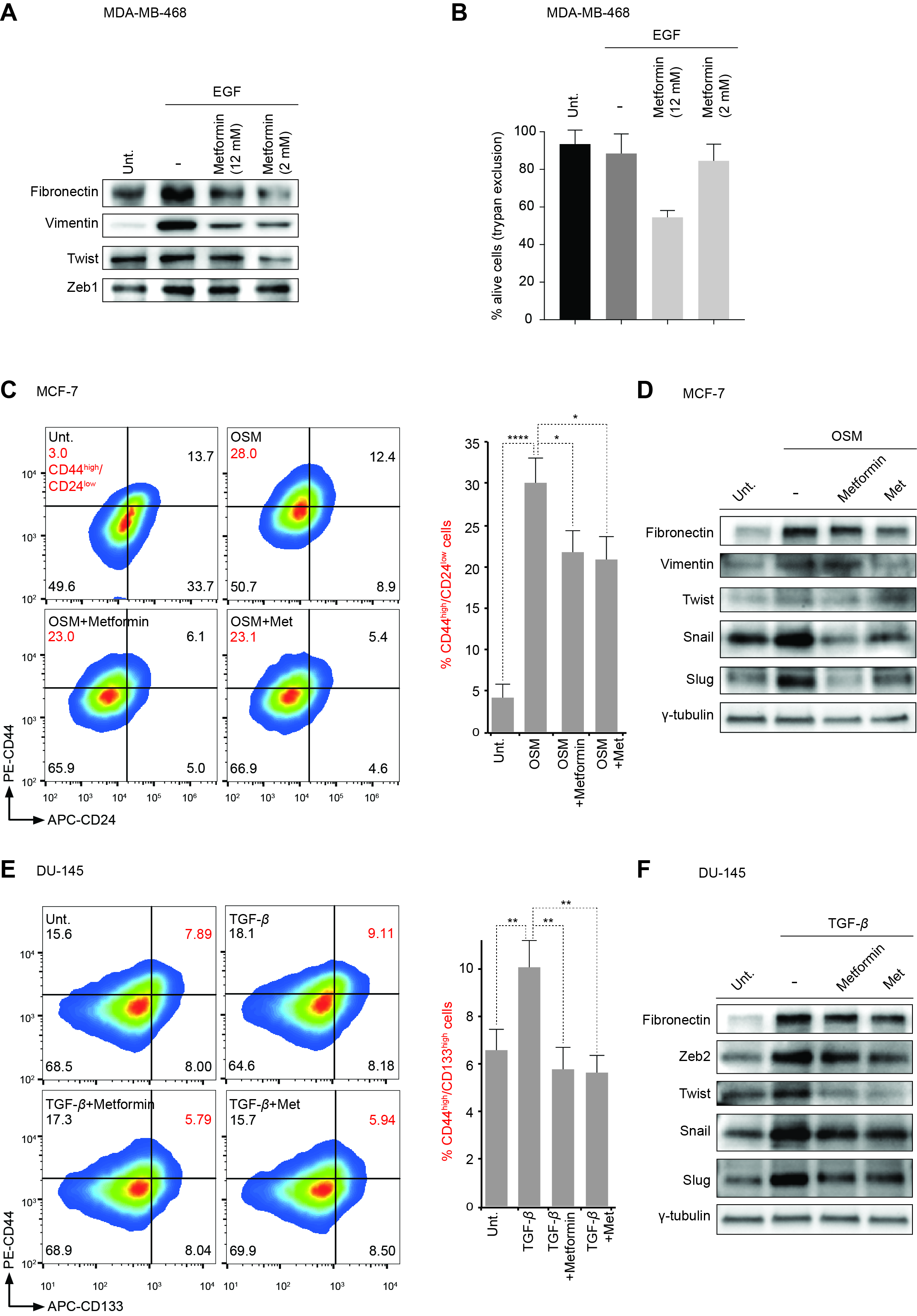

Supplement: S13 Fig — (A) Western blot analysis of mesenchymal markers and EMT-TF in MDA-MB-468 breast cancer cells treated as indicated for 72 h. (B) Bar chart of viable cells using Trypan blue exclusion of MDA-MB-468 breast cancer cells treated as indicated for 72h. (C) Flow cytometry analysis of cells surface markers of MCF-7 cells treated as indicated for 72 h and corresponding quantification. Bars and error bars, mean values and SD of three independent biological replicates. (D) Western blot analysis of mesenchymal markers and EMT-TF in MCF-7 breast cancer cells treated as indicated for 72 h. (E) Flow cytometry analysis of cells surface markers of DU-145 cells treated as indicated for 72 h and corresponding quantification. Bars and error bars, mean values and SD of three independent biological replicates. (F) Western blot analysis of mesenchymal markers and EMT-TF in DU-145 prostate cancer cells treated as indicated for 72 h. (TIF) [file pone.0206764.s013.tif]
