# Supplementary material for: Metformin reveals a mitochondrial copper addiction of mesenchymal cancer cells
Source: PLoS One. 2018 Nov 6;13(11):e0206764. doi: 10.1371/journal.pone.0206764 (PMC6219783; doi:10.1371/journal.pone.0206764)
Supplement: S14 Fig — (PDF) [file pone.0206764.s014.pdf]

S14 Fig. Sunthesis Supporting Information

Syntheses of Cu(I), Cu(II) and Fe(II) selective fluorescent probes

Rhodamine B-[(1,10-phenanthrolin-5-yl)aminocarbonyl]benzyl ester (RPA)

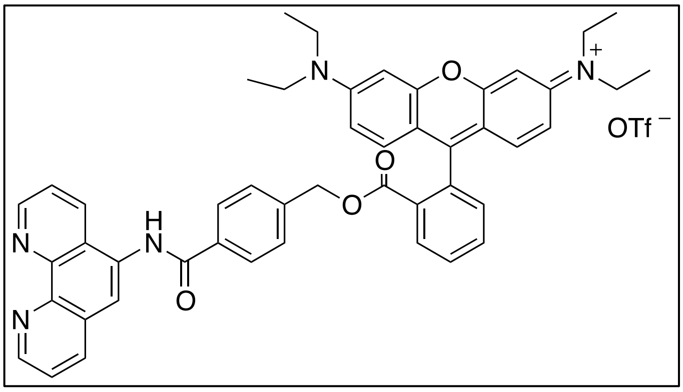

The mitochondrial iron (II) indicator RPA was prepared as described in the literature (Reference 26).

^{1}H NMR (300 MHz, $CDCl_3$) δ 11.53 (s, 1H), 9.36 – 9.16 (m, 2H), 8.85 (d, J = 8.1 Hz, 1H), 8.48-8.31 (m, 3H), 8.24 – 8.10 (m, 2H), 7.84 – 7.66 (m, 4H), 7.20-7.18 (m, 1 H), 7.06 – 6.89 (m, 6H), 6.73 (d, J = 9.3 Hz, 2H), 4.93 (s, 2H), 3.80 – 3.34 (m, 8H), 1.29 (t, J = 7.1 Hz, 12H).

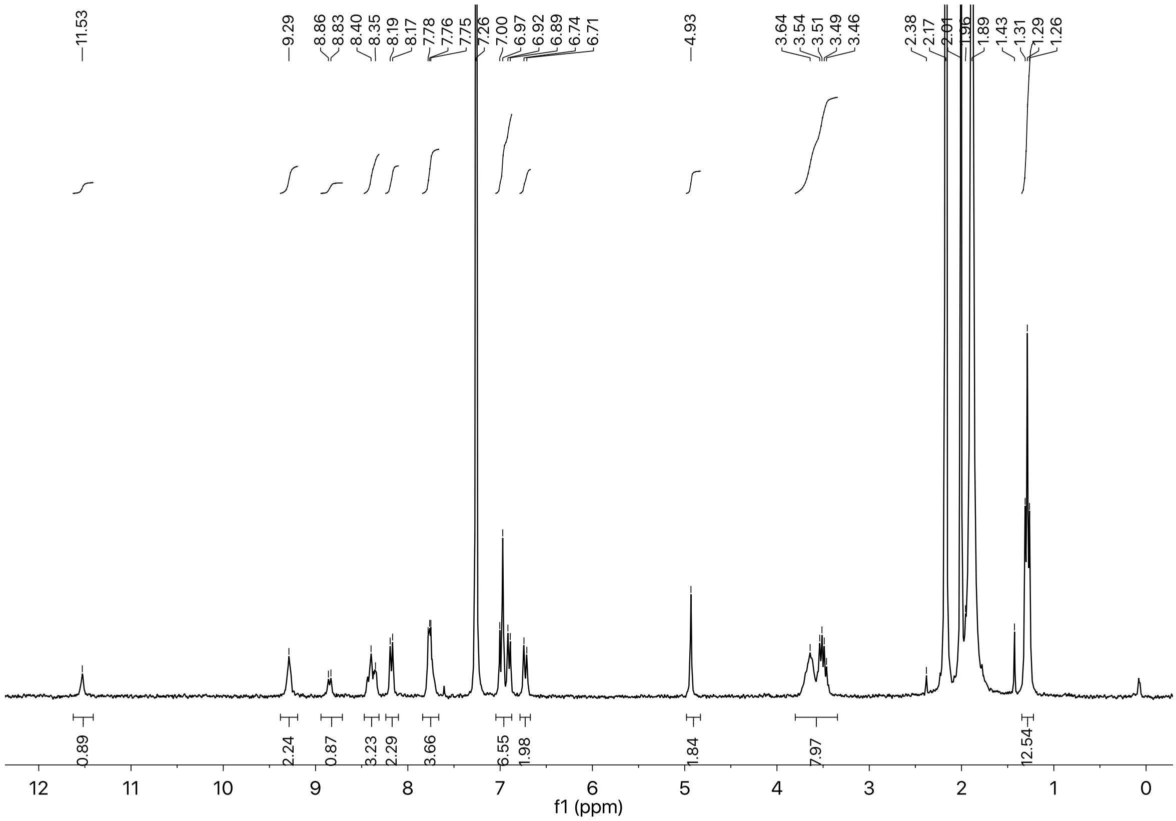

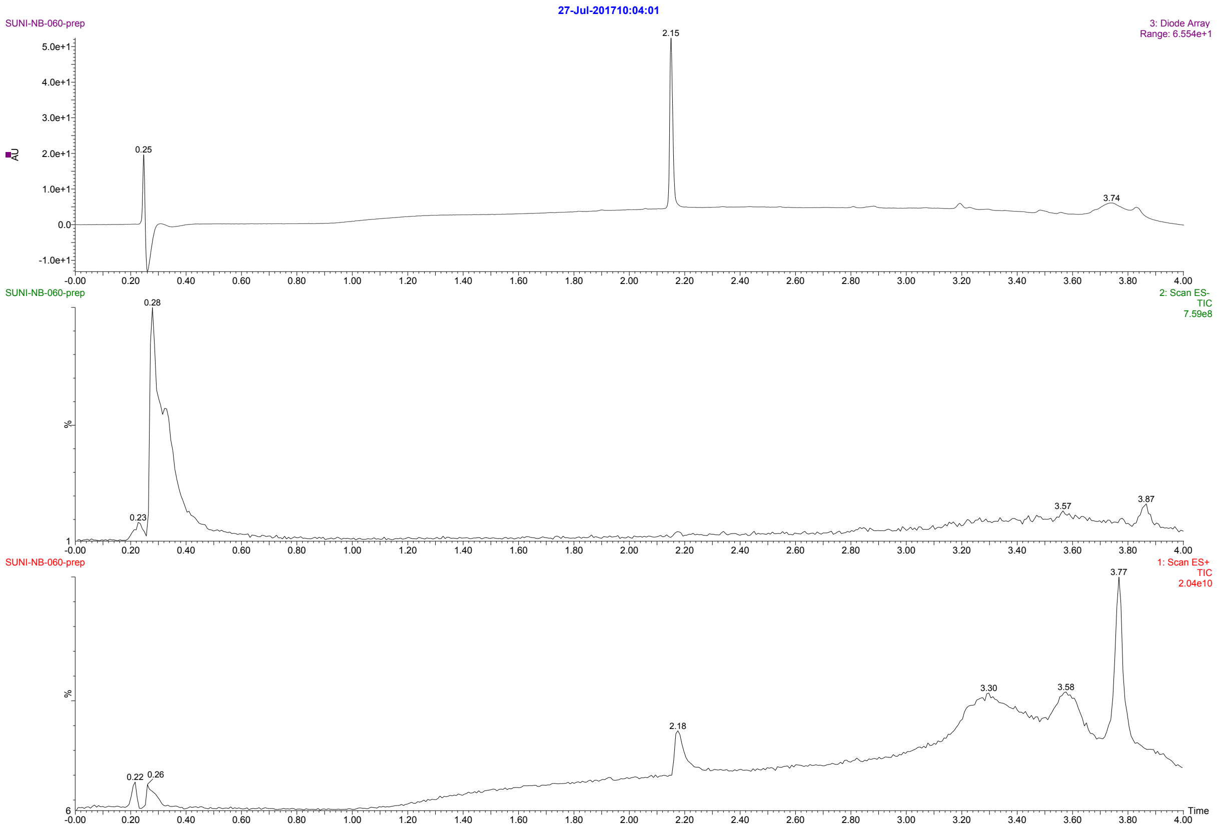
27-Jul-201710:04:01
SUNI-NB-060-prep
3: Diode Array
Range: 6.554e+1
AU
5.0e+1
4.0e+1
3.0e+1
2.0e+1
1.0e+1
0.0
-1.0e+1
0.25
2.15
3.74
SUNI-NB-060-prep
2: Scan ES-
TIC
7.59e8
%
0.28
0.23
3.57
3.87
SUNI-NB-060-prep
1: Scan ES+
TIC
2.04e10
%
0.22
0.26
2.18
3.30
3.58
3.77
Time
-0.00
0.20
0.40
0.60
0.80
1.00
1.20
1.40
1.60
1.80
2.00
2.20
2.40
2.60
2.80
3.00
3.20
3.40
3.60
3.80
4.00

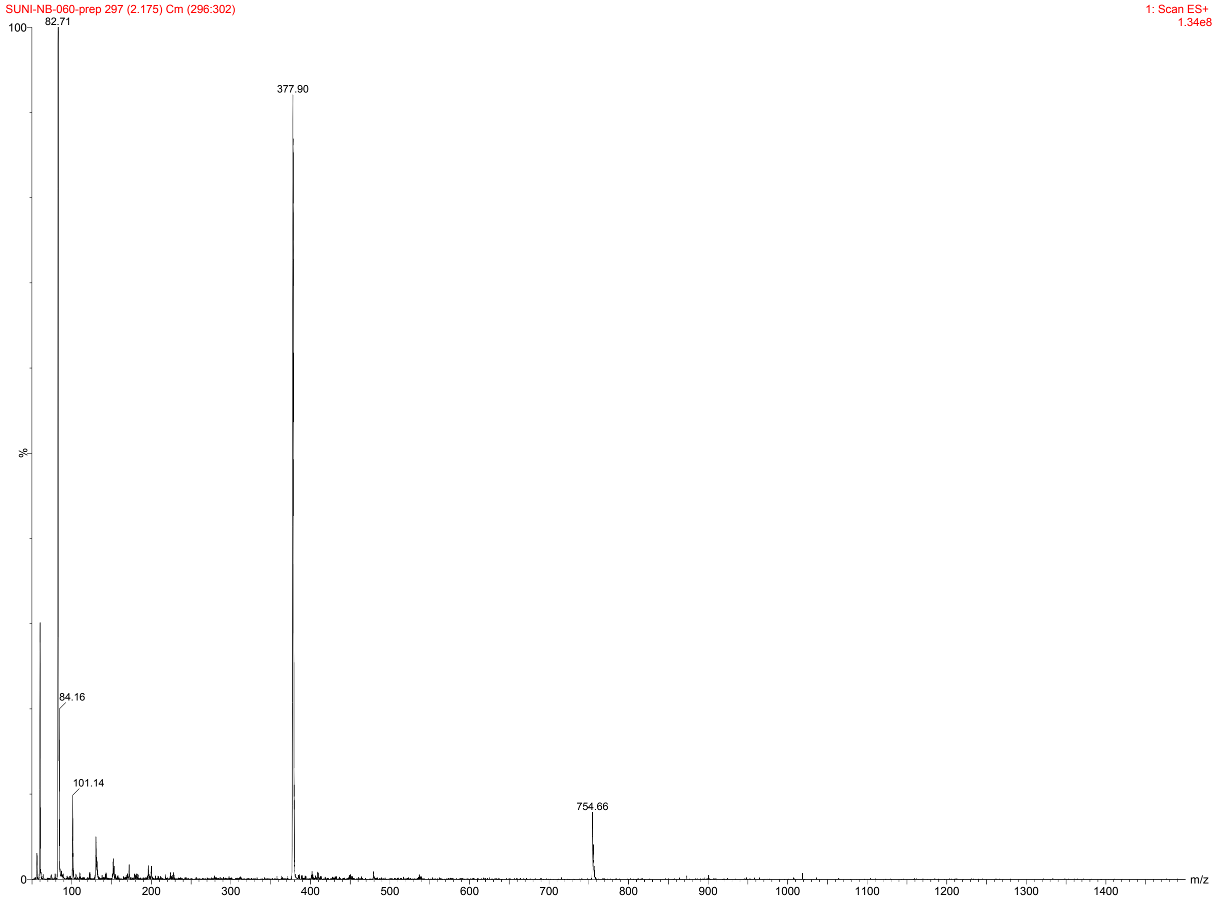
SUNI-NB-060-prep 297 (2.175) Cm (296:302)
1: Scan ES+
1.34e8
100
%
0
82.71
377.90
84.16
101.14
754.66
m/z
100
200
300
400
500
600
700
800
900
1000
1100
1200
1300
1400

Synthesis of (*E*)-2-(4-(bis(pyridin-2 ylmethyl)amino)styryl)-1,3,3-trimethyl-3*H*-indol-1-ium iodide:

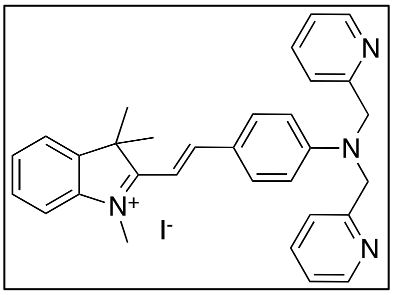

The mitochondrial Cu (II) probe was prepared as described in the literature (Reference 24).

^{1}H NMR (400 MHz, CD_3OD) δ 8.56 (ddd, *J* = 4.9, 1.8, 1.0 Hz, 2H), 8.28 (d, *J* = 15.9 Hz, 1H), 7.88 (d, *J* = 9.1 Hz, 2H), 7.81 (td, *J* = 7.7, 1.7 Hz, 2H), 7.66 (td, *J* = 7.0, 1.4 Hz, 2H), 7.60 – 7.49 (m, 2H), 7.39 (d, *J* = 7.9 Hz, 2H), 7.35 (ddd, *J* = 7.6, 4.9, 1.2 Hz, 2H), 7.26 (d, *J* = 15.8 Hz, 1H), 6.92 (d, *J* = 9.0 Hz, 2H), 5.07 (s, 4H), 4.00 (s, 3H), 1.79 (s, 6H).

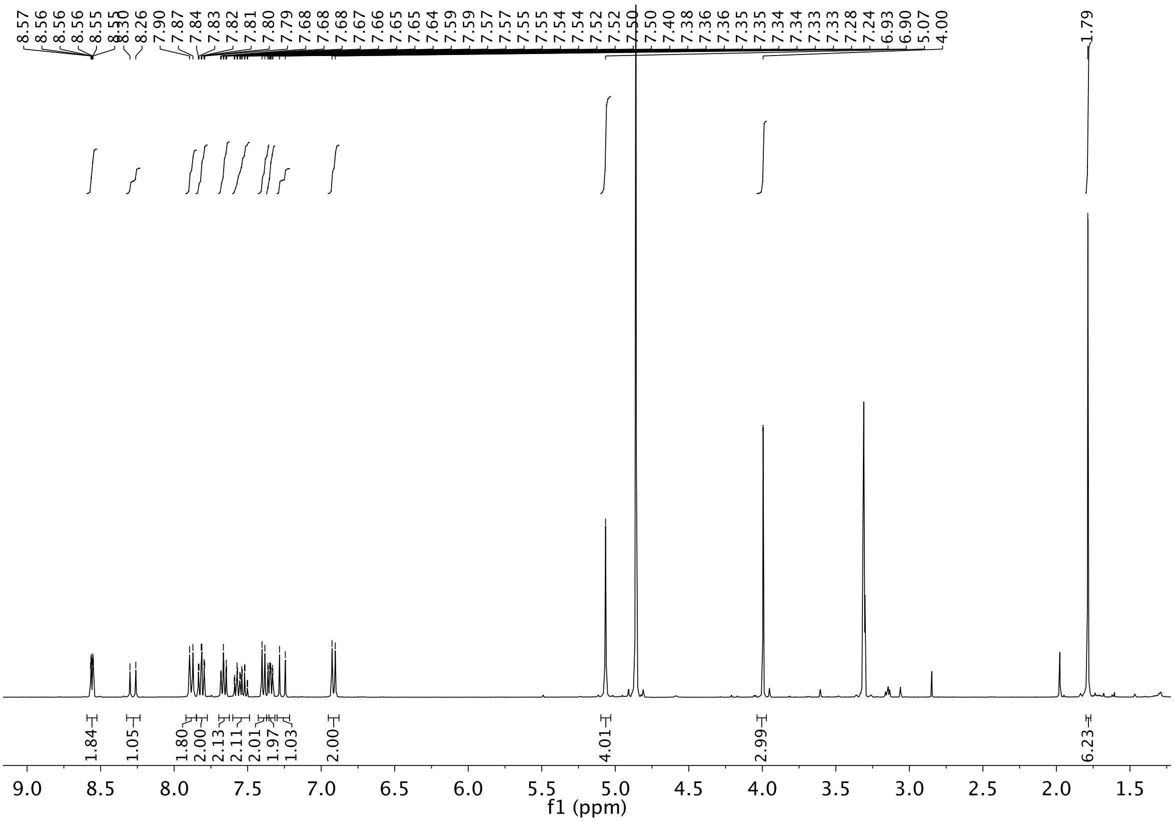

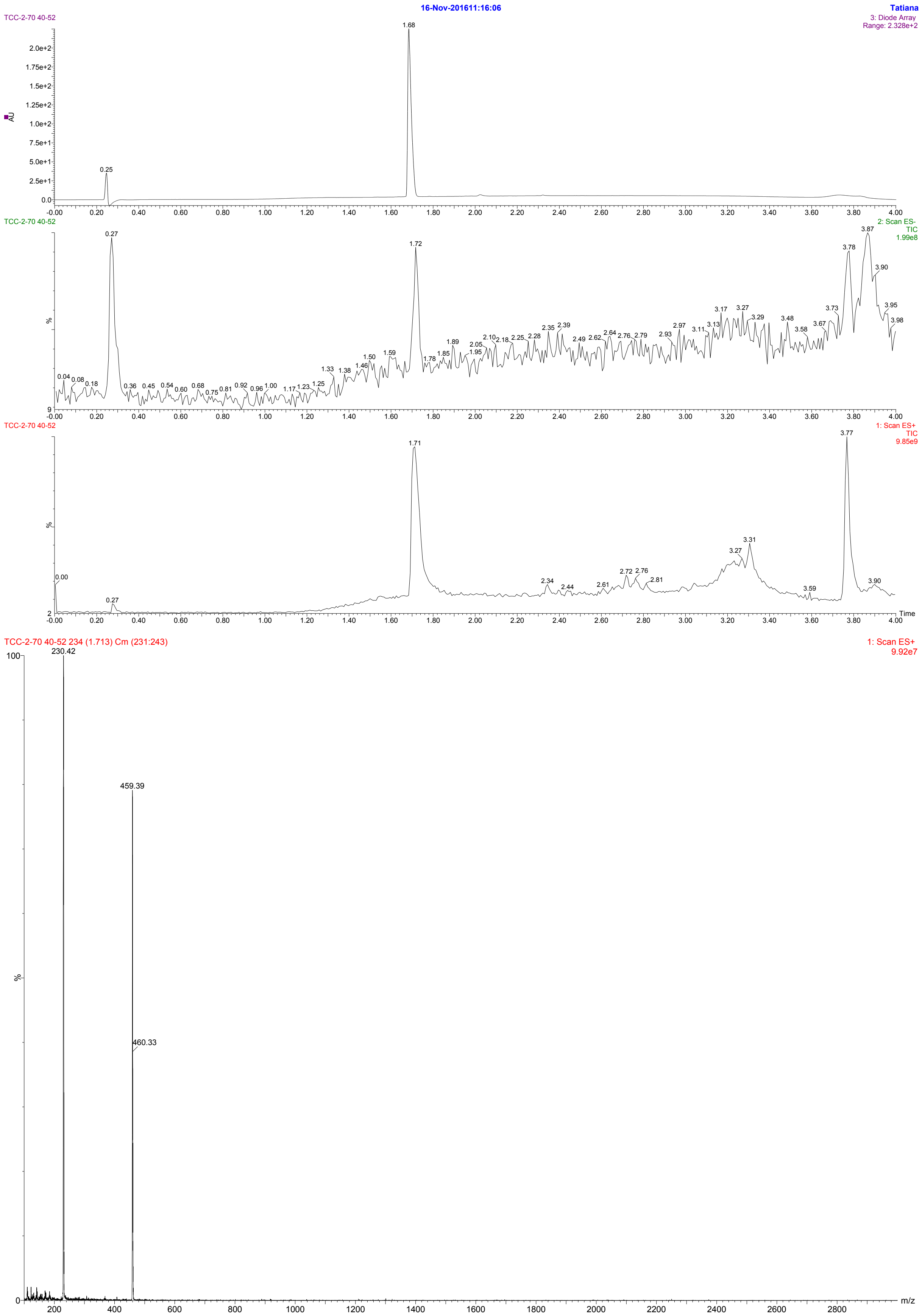
16-Nov-201611:16:06
Tatiana
TCC-2-70 40-52
3: Diode Array
Range: 2.328e+2
AU
1.68
0.25
TCC-2-70 40-52
2: Scan ES-
TIC
1.99e8
0.27
1.72
3.87
3.78
TCC-2-70 40-52
1: Scan ES+
TIC
9.85e9
1.71
3.77
3.31
Time
TCC-2-70 40-52 234 (1.713) Cm (231:243)
1: Scan ES+
9.92e7
230.42
459.39
460.33
%
m/z

Mito CS1

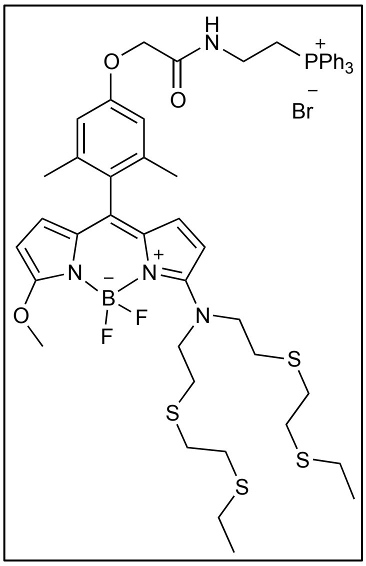

The mitochondrial Cu (I) sensor was synthesized following the experimental procedures described in the literature (Reference 25).

^{1}H NMR (500 MHz, CD_3OD) δ 7.94 – 7.83 (m, 10H), 7.77 (tdd, *J* = 7.3, 3.6, 1.9 Hz, 6H), 6.77 (s, 2H), 6.46 (d, *J* = 4.9 Hz, 1H), 6.24 (d, *J* = 5.0 Hz, 1H), 6.04 (d, *J* = 4.1 Hz, 1H), 5.73 (d, *J* = 4.0 Hz, 1H), 4.42 (s, 2H), 4.03 – 3.96 (m, 4H), 3.94 (s, 3H), 3.78 – 3.64 (m, 2H), 2.94 – 2.87 (m, 3H), 2.86 – 2.80 (m, 2H), 2.79 – 2.72 (m, 4H), 2.58 (q, *J* = 7.3 Hz, 4H), 2.11 (s, 6H), 1.22 (t, *J* = 7.4 Hz, 7H).

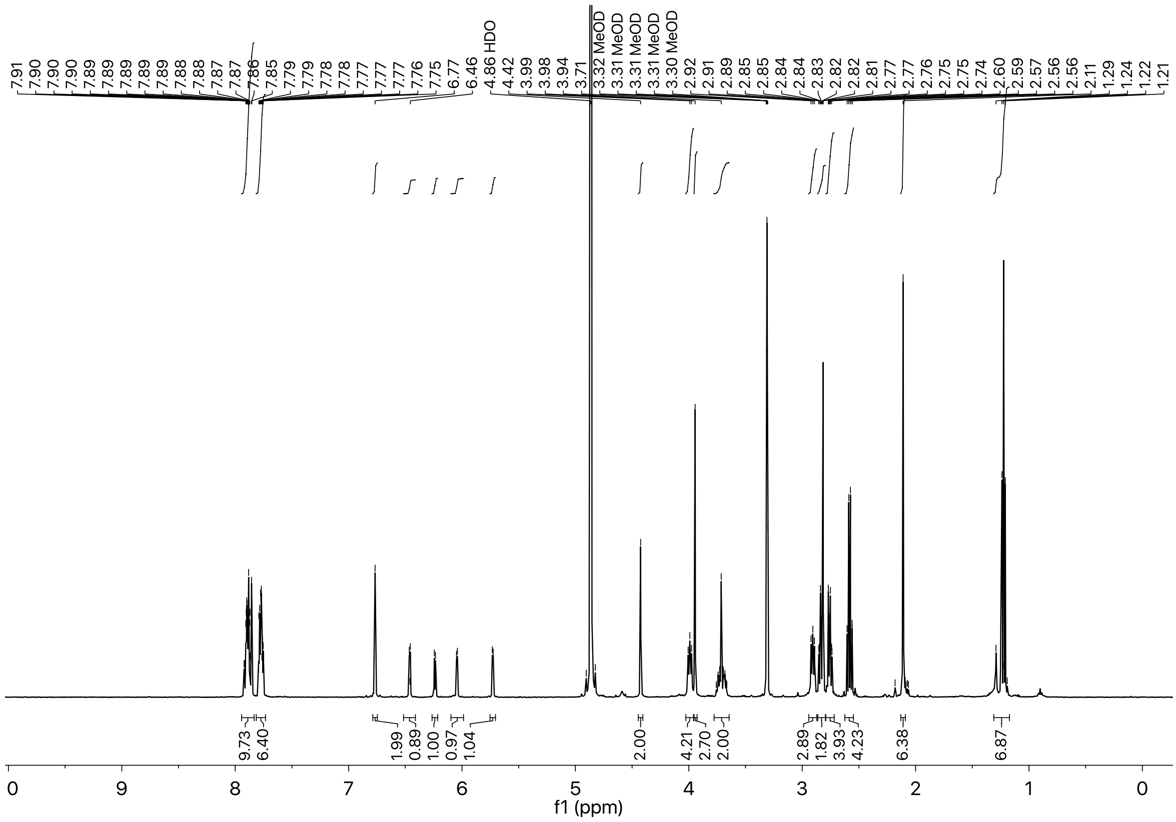
f1 (ppm)

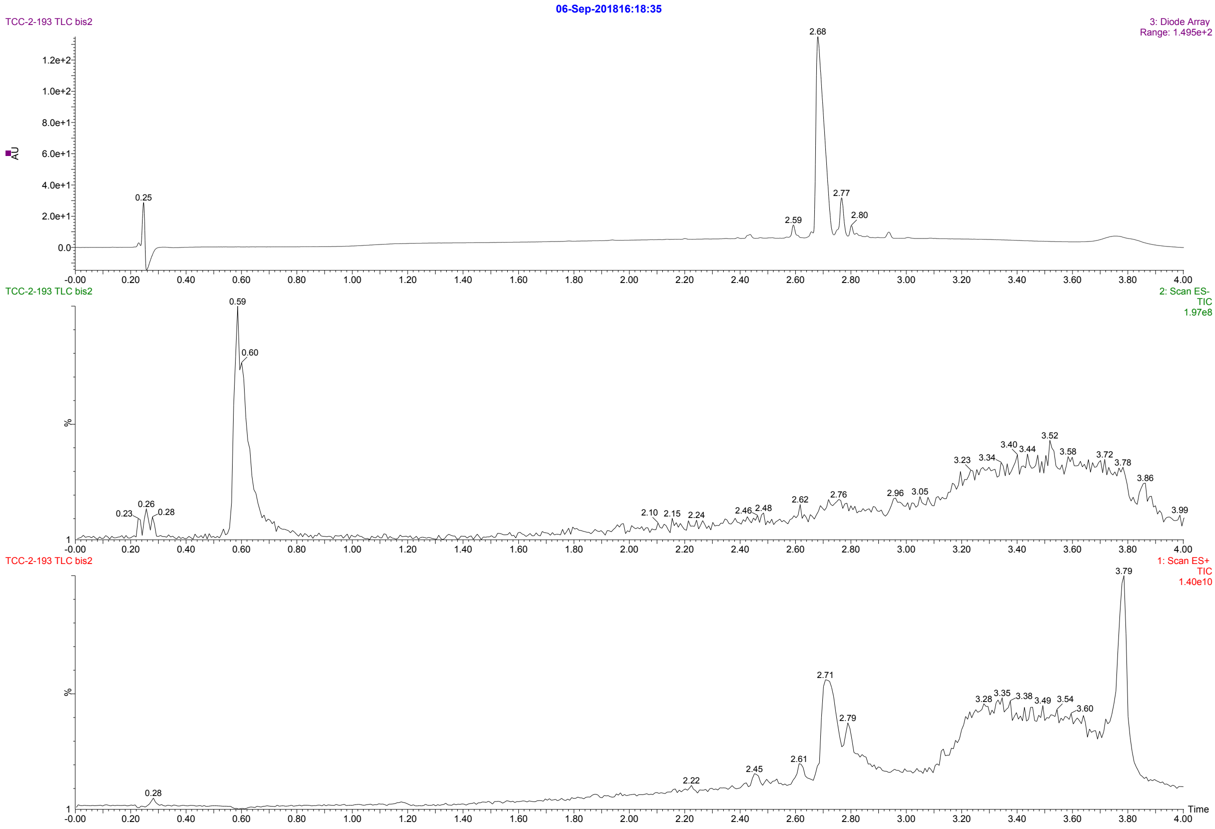
TCC-2-193 TLC bis2
06-Sep-201816:18:35
3: Diode Array
Range: 1.495e+2
2: Scan ES-
TIC
1.97e8
1: Scan ES+
TIC
1.40e10
Time

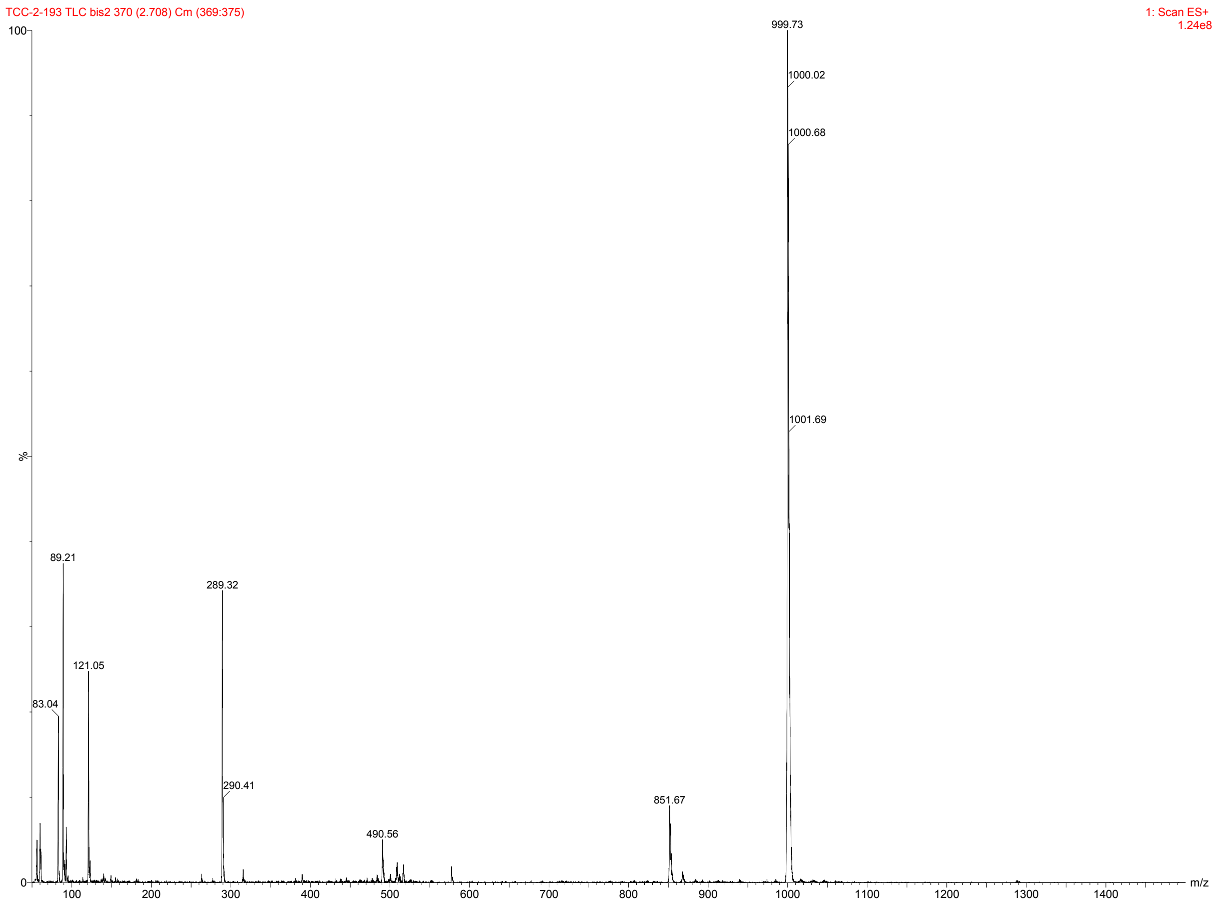
TCC-2-193 TLC bis2 370 (2.708) Cm (369:375)
1: Scan ES+
1.24e8
100
%
0
999.73
1000.02
1000.68
1001.69
89.21
289.32
121.05
83.04
290.41
851.67
490.56
m/z
100
200
300
400
500
600
700
800
900
1000
1100
1200
1300
1400
